# REMCARE: Pragmatic Multi-Centre Randomised Trial of Reminiscence Groups for People with Dementia and their Family Carers: Effectiveness and Economic Analysis

**DOI:** 10.1371/journal.pone.0152843

**Published:** 2016-04-19

**Authors:** Robert T. Woods, Martin Orrell, Errollyn Bruce, Rhiannon T. Edwards, Zoe Hoare, Barry Hounsome, John Keady, Esme Moniz-Cook, Vasiliki Orgeta, Janice Rees, Ian Russell

**Affiliations:** 1 Dementia Services Development Centre Wales, Bangor University, Bangor, Gwynedd, United Kingdom; 2 Institute of Mental Health, University of Nottingham, Nottingham, United Kingdom; 3 Bradford Dementia Group, University of Bradford, Bradford, United Kingdom; 4 Centre for Economics and Policy in Health, Bangor University, Bangor, Gwynedd, United Kingdom; 5 North Wales Organisation for Randomised Trials in Health and Social Care, Bangor University, Bangor, Gwynedd, United Kingdom; 6 Centre for Economics and Policy in Health, Bangor University, Bangor, Gwynedd, United Kingdom; 7 School of Nursing, Midwifery and Social Work, University of Manchester, Manchester, United Kingdom; 8 Dementia Care Research, Faculty of Health & Social Care, University of Hull, Hull, United Kingdom; 9 Division of Psychiatry, University College London, London, United Kingdom; 10 Clinical Psychology, Aneurin Bevan Health Board, Ystrad Mynach Hospital, Ystrad Mynach, Caerphilly, United Kingdom; 11 West Wales Organisation for Rigorous Trials in Health, Centre for Health Information Research and Evaluation, Swansea University, Swansea, United Kingdom; University of Glasgow, UNITED KINGDOM

## Abstract

**Background:**

Joint reminiscence groups, involving people with dementia and family carers together, are popular, but the evidence-base is limited. This study aimed to assess the effectiveness and cost-effectiveness of joint reminiscence groups as compared to usual care.

**Methods:**

This multi-centre, pragmatic randomised controlled trial had two parallel arms: intervention group and usual-care control group. A restricted dynamic method of randomisation was used, with an overall allocation ratio of 1:1, restricted to ensure viable sized intervention groups. Assessments, blind to treatment allocation, were carried out at baseline, three months and ten months (primary end-point), usually in the person's home. Participants were recruited in eight centres, mainly through NHS Memory Clinics and NHS community mental health teams. Included participants were community resident people with mild to moderate dementia (DSM-IV), who had a relative or other care-giver in regular contact, to act as informant and willing and able to participate in intervention. 71% carers were spouses. 488 people with dementia (mean age 77.5)were randomised: 268 intervention, 220 control; 350 dyads completed the study (206 intervention, 144 control). The intervention evaluated was joint reminiscence groups (with up to 12 dyads) weekly for twelve weeks; monthly maintenance sessions for further seven months. Sessions followed a published treatment manual and were held in a variety of community settings. Two trained facilitators in each centre were supported by volunteers. Primary outcome measures were self-reported quality of life for the person with dementia (QoL-AD), psychological distress for the carer (General Health Questionnaire, GHQ-28). Secondary outcome measures included: autobiographical memory and activities of daily living for the person with dementia; carer stress for the carer; mood, relationship quality and service use and costs for both.

**Results:**

The intention to treat analysis (ANCOVA) identified no differences in outcome between the intervention and control conditions on primary or secondary outcomes (self-reported QoL-AD mean difference 0.07 (-1.21 to 1.35), F = 0.48, p = 0.53). Carers of people with dementia allocated to the reminiscence intervention reported a significant increase in anxiety on a General Health Questionnaire-28 sub-scale at the ten month end-point (mean difference 1.25 (0.25 to 2.26), F = 8.28, p = 0.04). Compliance analyses suggested improved autobiographical memory, quality of life and relationship quality for people with dementia attending more reminiscence sessions, however carers attending more groups showed increased care-giving stress. Economic analyses from a public sector perspective indicated that joint reminiscence groups are unlikely to be cost-effective. There were no significant adverse effects attributed to the intervention. Potential limitations of the study include less than optimal attendance at the group sessions—only 57% of participants attended at least half of the intervention sessions over the 10 month period, and a higher rate of study withdrawal in the control group.

**Conclusions:**

This trial does not support the clinical effectiveness or cost-effectiveness of joint reminiscence groups. Possible beneficial effects for people with dementia who attend sessions as planned are offset by raised anxiety and stress in their carers. The reasons for these discrepant outcomes need to be explored further, and may necessitate reappraisal of the movement towards joint interventions.

**Trial Registration:**

ISRCTN Registry ISRCTN42430123

## Introduction

There is increasing recognition that in dementia care psychosocial interventions may have comparable value to pharmacological approaches [1,2] and may be preferable in some contexts, e.g. where medication may be ineffective or have negative side-effects [3,4], as long as there is no additional strain on family carers [5]. Despite reminiscence being the most popular therapeutic approach to working with people with dementia, there is little evidence on effectiveness and cost-effectiveness. Reminiscence work with people with dementia has an extensive history [6], engendering enjoyable activities that promote communication and well-being. Reminiscence works with early memories, often intact in dementia, drawing on preserved abilities, rather than emphasising impairments. A Cochrane review on reminiscence therapy for people with dementia [7] did not identify any rigorous trials or economic analyses in this field. All four randomised controlled trials (RCTs) suitable for analysis were small or of poor quality and each examined different types of reminiscence work. One of the two studies that were group-based included family carers in the groups, in line with a meta-analysis on interventions with family carers of people with dementia suggesting that joint approaches may be more effective in improving carer outcomes than approaches targeted only at the carer [5]. The trials together identified significant improvements in cognition and mood 4–6 weeks after treatment, and reduced stress in carers participating with the person with dementia in a reminiscence group. Recent reminiscence research [8–10] has not involved family carers with people with dementia, making use of technology-based reminiscence or evaluating groups in a care home context. The National Institute for Health and Care Excellence and Social Care Institute for Excellence (NICE-SCIE) Guideline on dementia [3] found insufficient evidence to recommend reminiscence therapy, although its potential impact on mood of the person with dementia was highlighted. The aim of our REMCARE trial was to evaluate a specific, promising form of reminiscence work, avoiding the methodological limitations of previous work. Thus we set out to assess the effectiveness and cost-effectiveness of joint reminiscence groups for both people with dementia and their carers as compared with usual care.

## Methods

### Design

**Fig 1:**
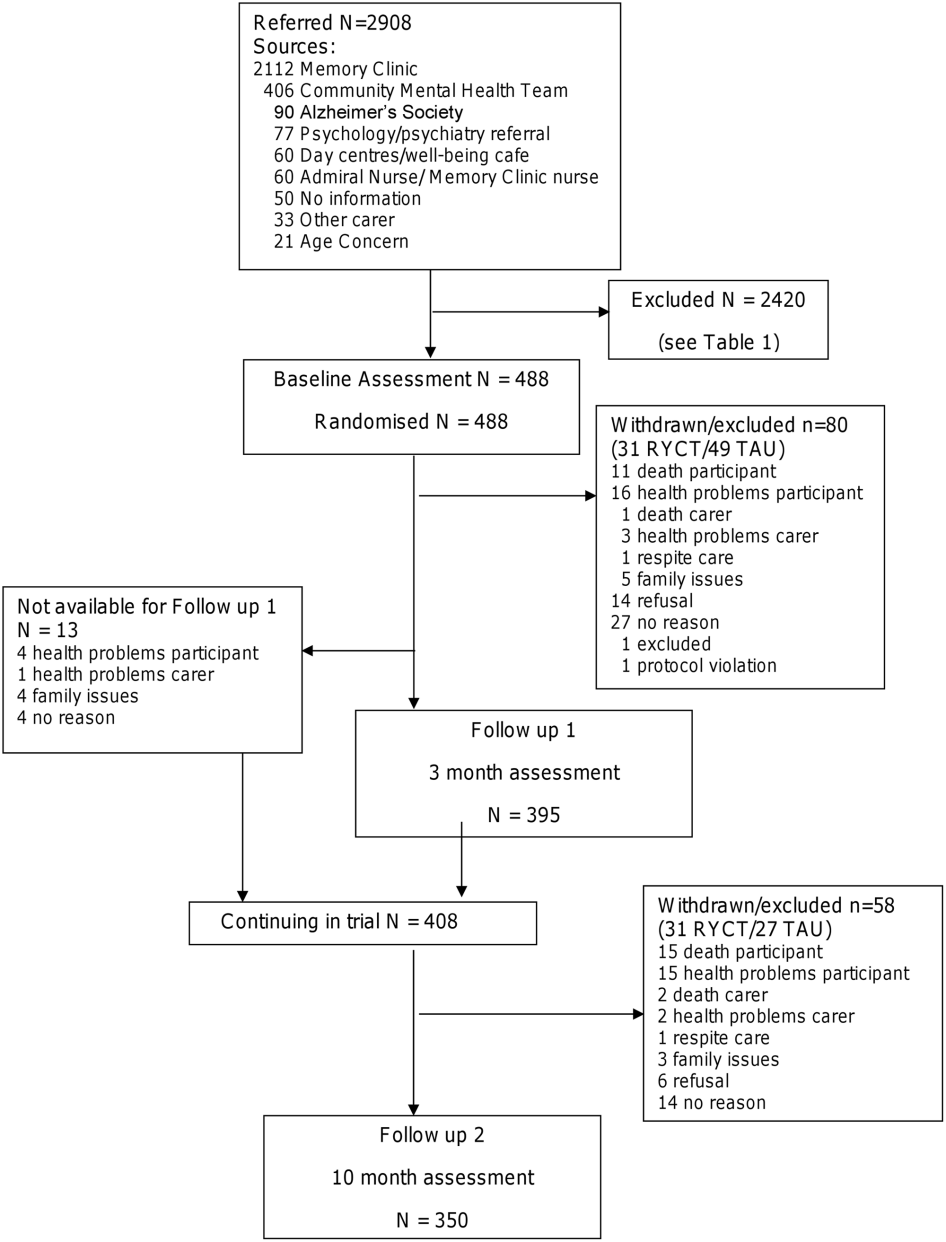
Consort diagram of participant flow through study.

The design was a pragmatic eight-centre randomised trial (Fig 1) with two parallel arms: an intervention group, and a control group receiving care as usual. Assessments, blind to treatment allocation, were carried out at baseline, three months and ten months (the primary end point). Randomisation used a dynamic allocation method [11] stratifying for spousal or non-spousal relationship of the dyad. Complete list randomisation for each wave of recruitment within each centre was completed. Randomisation was carried out remotely by the NWORTH accredited Clinical Trials Unit, initiated by a local researcher who did not take part in follow-up assessments. This researcher arranged for those pairs (up to 12) randomised to the intervention group to attend sessions, and liaised with the group facilitator. Though participants could not be blinded to their allocated treatment, all follow-up data were gathered by blinded interviewers. In order to reduce the risk of participants occasionally and inadvertently unblinding researchers, explicit reminders were given to participants before assessment visits and self-report measures were used wherever feasible. Assessors were also asked to record their impression of the arm to which each participant belonged so that any bias could be detected. A favourable ethical opinion was given by the Multi-centre Research Ethics Committee for Wales (ref no. 07/MRE09/58).

### Participants

Recruitment took place through mental health services for older people in each area especially Memory Clinics and Community Mental Health Teams for Older People, associated day services and voluntary sector agencies such as the Alzheimer’s Society, Dementia UK and Age Concern in the areas Bangor, Bradford, London, Manchester, Newport and Hull. Recruitment took place in three to five waves in each centre, between June 2008 and July 2010. Assessments were usually carried out in the person‘s home, and treatment groups held in a variety of community settings. Participants were 488 people (mean age 77.5) with dementia initially living in the community. Most carers were spouses (71%). 350 dyads completed the study. Participants were free to seek additional assistance and support elsewhere at any time after baseline.

### Inclusion criteria

To be included in the study, participants had to: have mild/moderate dementia diagnosed using DSM-IV criteria; be able to communicate and understand communication, to some degree (score of 1 or 0 on specific items of the Clifton Assessment Procedures for the Elderly—Behaviour Rating Scale [12]); be able to engage in group activity; live in the community; have a relative or other carer who could act as an informant, and was willing and able to participate in the joint intervention; not have any serious problem which could undermine participation (e.g. major physical or sensory disabilities, learning disability, or severe agitation).

### Intervention

The intervention followed the ‘Remembering Yesterday, Caring Today’ (RYCT) manual [13], developed in the trial platform for this study [14]. Joint reminiscence groups emphasise active and passive reminiscence by both carers and people with dementia. Group sessions were held weekly over 12 consecutive weeks, followed by seven monthly maintenance group sessions. Sessions were led by two trained facilitators in each centre, supported by trained volunteers. The manual recommends a blend of activities for each session, based on core principles. Each session lasted two hours and focused on a different theme, including childhood, schooldays, working life, marriage, and holidays and journeys. Dyads were encouraged to contribute with materials brought from home. Maintenance sessions followed a similar pattern. Each session blended work in large and small groups, and a range of activities including art, cooking, physical re-enactment of memories, singing and oral reminiscence. Since inclusion of the person with dementia was considered paramount, facilitators and volunteers guided carers to value their contribution by allowing the person with dementia to actively participate. Fidelity of the intervention was assured through regular meetings of the treatment facilitators with the trainer, and by the completion of an adherence checklist at the end of each group session.

### Usual care (treatment as usual—TAU)

The services and interventions available to people with dementia and family carers randomised to receive usual treatment varied between and within centres and over time. Both this group and the treatment group could access any other services, except when the reminiscence groups were scheduled at the same time as an alternative activity.

### Ethical arrangements

No harmful side-effects from participating in reminiscence groups have been documented [7]. Participants entered the study only after giving written informed consent in accordance with the provisions of the Mental Capacity Act 2005. In the event of a participant being judged to lose capacity to consent to participate during the trial, the views of a personal consultee (the carer) were sought regarding continuation. At any point where a participant with dementia became distressed by the assessments they were discontinued. Informed consent was sought separately from the family carer for their own participation.

### Outcome measures

Primary and secondary measures were completed at baseline, three months after baseline (first follow-up) and ten months after baseline (second follow-up and primary end-point). For the first follow-up the interviews were conducted after the completion of the weekly reminiscence sessions, and for the second follow-up they were after the final monthly maintenance session.

#### Primary outcome measures

a) Quality of life of the person with dementia, using the scale *Quality of Life in Alzheimer’s Disease* (QoL-AD), which covers 13 domains of quality of life [15,16]. For this measure, up to two missing values may be replaced with the mean of the other scores. Scores range from 13 to 52; higher scores indicate better quality of life.

b) Carer’s mental health, assessed using the 28-item, self-completed *General Health Questionnaire* GHQ-28 [17] which has been widely used in care-giver research [18,19] and comprises 4 sub-scales: anxiety/insomnia, depression, social dysfunction and somatic symptoms. Higher scores indicate worse mental health, ranging from 0 to 84.

#### Secondary outcome measures

a) Autobiographical memory, assessed using an extended version of the *Autobiographical Memory Interview* (AMI) [20] assessing recall of personal memories with sub-scales for both factual (semantic) information (e.g. names of schools or teachers) and specific incidents. The scores range from 0 to 108 for semantic subscale and 0 to 39 for incidents. Higher scores indicate better memory.

b) Quality of relationship, assessed separately by both person with dementia and carer using the *Quality of the Carer–Patient Relationship* (QCPR) [21] comprising 14 items rated on 5-point Likert scales, with two sub-scales designed to assess the warmth of the relationship and the absence of conflict and criticism. Scores range from 14–70 with higher scores reflecting better relationships.

c) Depression and anxiety, using the *Cornell Scale for Depression in Dementia* (CSDD) [22] and the *Rating Anxiety In Dementia* (RAID) [23] for the person with dementia. The CSDD (19 items) and the RAID (18 items) are based on interviews with the people with dementia and their carers. The CSDD scores range from 0 to 38 with higher scores reflecting a higher level of depression. The *Hospital Anxiety and Depression Scale* (HADS) [24] (used for carers) is a well-validated 14-item, self-completed scale with sub-scales for anxiety and depression suitable for use with adults of all ages. For each HADS sub-scale, a single missing value may be replaced with the mean of the other scores from that sub-scale. Each subscale is scored on a 0 to 21 range with higher scores indicating elevated levels of anxiety or depression.

d) Stress specific to care-giving, using the *Relative's Stress Scale* [25], which asks the care-giver to complete 15 5-point Likert items. Scores range is 0 to 60 with higher scores indicating a higher level of stress.

e) Quality of life of person with dementia, rated by the care-giver using the proxy version of the *QoL-AD* [15], identical in structure and content to the version completed by the person with dementia. Scores range from 13 to 52 with higher scores indicating a better perceived quality of life.

f) General quality of life of both care-giver and person with dementia, used the 5 item *EQ-5D-3L* and associated visual analogue scale *EQ VAS* [26]. Carers completed the measure from their own perspective and for the person with dementia, who would also complete it whenever possible.

g) Functional ability of person with dementia, using the *Bristol Activities of Daily Living Scale* [27], a 20-item scale completed by the carer. Scores range from 0 to 60 with a higher score reflecting more dependency.

h) Use of health, social care and voluntary services by the person with dementia and their carer was recorded using an adapted Client Services Receipt Inventory (CSRI) [28].

### Micro-costing of reminiscence group therapy and maintenance

A micro-costing of the RYCT intervention was undertaken by recording the types and quantities of resource input including: staff time, materials, room rental, training and supervision of staff. Information was provided by the principal investigators for each site, who oversaw the resources available for the intervention and by group facilitators in relation to staff time utilisation, materials and other resources used. Unit costs, tariffs or prices were then assigned for each item as appropriate, with actual costs used for items such as transport, venue hire and freelance facilitator and trainer fees. Appropriate national salary scales were used for staff who were NHS or University employees, including national insurance and pension costs, but not organisational overheads or high-cost area supplements. The costs for each RYCT programme of 12 weekly and 7 maintenance sessions for which complete data were available were calculated, and the mean cost per programme and for each expenditure category was then derived. The cost per dyad was then calculated by dividing the cost per programme by the number of dyads randomised to the programmes for which the costing information was available.

### Analysis

The trial was initially powered to detect a standardised difference of 0.38 in the QoL-AD rated by the person with dementia and 0.28 in the General Health Questionnaire-28 or carer-rated QoL-AD, requiring 200 dyads in each arm to complete the 10 month assessment. This allowed for clustering effects within groups, using an intraclass correlation coefficient (ICC) of 0.1. With predicted attrition of 30% the initial target sample size was 576 dyads. During the trial this target was revised in the light of lower clustering effects (ICC calculated at 0.02) and better retention rates. Assuming 72% retention, the revised recruitment target of 508 provided a potential sample of 366 at 10 month follow-up. This provided 80% power to detect a standardised difference of 0.30 in the primary outcome measures at the 5% significance level. Intention to treat analysis was used primarily. Multiple imputation methods were used to estimate missing outcome measure scores at a time point, using a linear regression model accounting for age, gender, spousal care, centre, wave and other outcome scores. At follow up time points the treatment group allocation was also included in the model. For baseline, one imputation was used due to the small amount of missing outcomes; at follow up time points five imputations were used. The number of imputations used reflected the percentage of missing data present in the dataset [29]. The primary model fitted was an ANCOVA using 10-month outcome as the dependent variable, baseline score on the outcome measure and the age of the person with dementia as covariates, treatment allocation, gender of the person with dementia, spousal (spouse/other) as fixed factors and location and wave as random factors, with the interaction between location and allocation also being taken into account. Carer age and gender were also added for carer and proxy outcomes.

Secondary analyses included analysis of the secondary outcomes at the 10 month point and also fitted all the models using the 3-month outcome as the dependent variable. A further compliance analysis was also undertaken including a variable denoting the number of sessions that participants assigned to the intervention arm had actually participated in. SPSS PASW (version 18) was used for the analyses.

### Economic Analyses

#### Cost-effectiveness analysis

The economic analysis takes a multi-sectoral public sector perspective spanning the NHS (dementia services, primary and secondary care) and local government. Cost-effectiveness was evaluated in terms of the primary outcome for the person with dementia (the disease specific QoL-AD) at the primary end-point, on an intention to treat basis, using only cases with full cost data. Missing QoL-AD values were imputed as described previously. The incremental cost effectiveness ratio (ICER) was calculated as the ratio of the difference in costs to the difference in outcomes between the intervention and control groups. This incremental cost effectiveness ratio point estimate reflects the mean cost of a one point change on the scale reflecting an improvement in quality of life. Non-parametric bootstrapping (5,000 replications) was used to address the uncertainty associated with point estimates of costs and outcomes, providing a confidence interval for the ICER. It was planned to produce cost effectiveness acceptability curves to provide probabilistic analysis for a range of cost effectiveness ceilings for policy makers.

#### Secondary Cost-Utility Analysis

A cost utility analysis was conducted, calculating costs per quality adjusted life year (QALY), with QALYs derived from the EQ-5D. The area under the curve method was used to generate total QALYs. Two analyses were carried out; the first using the EQ-5D as completed by the person with dementia and the second the self-report EQ-5D completed by their carers. Only cases with full cost data were included, but missing EQ-5D values were imputed as described above. It was planned to produce cost effectiveness acceptability curves with non-parametric bootstrapping using 5000 replications so that the probability of the intervention meeting a range of cost per QALY thresholds could be estimated.

## Results

### Demographic and follow up information

Across the study sites, 2908 potential participants were considered for inclusion, either through identification by screening of clinical notes or by referral from clinical teams or other services. Table 1 indicates the reasons for losses between referral / screening and randomization. The major reasons were related to it not proving possible to contact the person or carer or to the application of the inclusion/exclusion criteria. Of those contacted, eligible and available 36% agreed to take part. The final sample size of 350 dyads completing the 10 month end-point assessment represented 95% of the revised target sample size, with an overall attrition rate of 28%, or 22% excluding deaths (Fig 1). Of the people with dementia, 242 (50%) were female, 447 (95%) were of white ethnicity, 337 (72%) were married and mean age was 77.5 (sd 7.3). The carers comprised 325 (67%) females, 448 (96%) of white ethnicity, 345 (74%) were caring for their spouse and mean age was 69.7 (sd 11.6). Table 2 indicates that the people with dementia and carers in the two groups were well matched at baseline for clinical and demographic characteristics. Attrition was higher in the control group at 10 months (34% control v. 24% intervention) with those lost from the control group having a lower level of dementia severity. No serious adverse events related to the trial were recorded.

**Table 1:** Reasons for losses between referral and randomisation.

Reason	Total (%)
**Total referred or screened**	**2908**
Unable to find Memory Clinic record	115 (5)
Could not make contact by telephone	393 (16)
Does not wish to take part	863 (36)
Does not meet clinical criteria	108 (4)
No suitable carer	69 (3)
Now in residential care	95 (4)
Already participating in a similar study	14 (<1)
Exclusion criteria apply	91 (4)
Unable to attend on the day that joint reminiscence groups are being held	113 (5)
**Other**	
Family situation at the time	41 (2)
Carer or participant died	96 (4)
Health issues for participant or carer	168 (7)
Participant unaware of dementia diagnosis	5 (<1)
Not available	209 (9)
Does not like groups—reference to dislike of intervention	17 (<1)
Unknown	23 (1)
**Total lost between referral/screening and randomisation**	**2420**
**Total number randomised**	**488**
**Conversion rate**	**17%**

**Table 2:** Baseline characteristics of people with dementia and carers—mean (sd).

People with dementia	Possible range of scores	Intervention (n = 268)	Control (n = 219)
Age	-	77.5 (7.3)	77.3 (7.2)
Female	=	47%	52%
Married	=	72%	72%
Spousal relationship	=	70%	72%
Quality of Life in Alzheimer’s Disease (QoL-AD) (self rating)	13–52	37.5 (5.3)	37.0 (5.4)
Quality of Life in Alzheimer’s Disease (QoL-AD) (proxy rating)	13–52	31.5 (6.3)	31.5 (6.5)
Autobiographical Memory Interview—semantic (AMIF)	0–108	56.1 (23.0)	54.3 (24.2)
Autobiographical Memory Interview—incidents (AMIM)	0–39	12.5 (6.9)	12.9 (7.8)
Quality of Carer Patient Relationship (QCPR)	14–70	57.8 (6.4)	57.5 (6.1)
Bristol Activities of Daily Living Scale (BADLS)	0–60	16.6 (9.4)	15.1 (9.8)
Rating of Anxiety in Dementia (RAID)	0–54	8.8 (7.5)	8.2 (6.6)
Cornell Scale for Depression in Dementia (CSDD)	0–38	7.0 (4.9)	6.9 (5.1)
**Carers**		**Intervention (n = 268)**	**Control (n = 219)**
Age	-	69.6 (11.6)	69.7 (11.6)
Female	-	70%	63%
Married	-	87%	82%
General Health Questionnaire (GHQ28)	0–84	22.8 (11.7)	23.1 (12.0)
Hospital Anxiety & Depression Scale (HADS) Anxiety	0–21	6.4 (4.3)	6.0 (4.2)
Hospital Anxiety & Depression Scale (HADS) Depression	0–21	4.3 (3.5)	4.1 (3.4)
European Quality of Life 5 Dimensions Visual Analogue Scale (EQ-5D VAS)	0–100	74.3 (17.8)	72.9 (19.7)
Relatives Stress Scale (RSS)	0–60	21.8 (10.9)	21.3 (10.9)
Quality of Carer Patient Relationship (QCPR)	14–70	53.5 (8.8)	53.6 (8.6)

### Primary and secondary outcomes

The GHQ-28 was the only outcome measure showing deviation from a normal distribution and accordingly was subject to a natural log-transformation. The intention to treat analyses indicated there were no differences in outcome between the intervention and control conditions on primary outcomes at the ten-month end point (self-reported QoL-AD mean difference 0.07, s.e. 0.65;, p = 0.53; GHQ 28 mean difference 0.09, s.e. 0.06;, p = 0.35: Tables 3 and 4). There were also no differences on the primary outcomes at the three-month end point (self-reported QoL-AD mean difference -0.76, s.e. 0.59;, p = 0.63 (Table 3), GHQ mean difference 0.02, s.e. 0.05 p = 0.86 (Table 4)).

**Table 3:** Primary and secondary end point results adjusted analysis models for person with dementia measures. Adjusted for participant age, baseline outcome score, centre, participant gender, relationship (spousal/non-spousal).

Measure	Data	Primary end point (10 month follow up)							Secondary end point (3 month follow up)						
		Missing (N = 350)	Treatment Mean	Control Mean	Group difference Mean	95% CI of MD	P value	Effect Size	Missing (N = 395)	Treatment Mean	Control Mean	Group Difference Mean	95% CI of MD	P value	Effect Size
QoL-AD (self)	Complete case	46	36.86	36.79	0.07	(-1.20, 1.34)	0.53	0.01	34	36.88	37.64	-0.76	(-1.92, 0.4)	0.63	0.07
QoL-AD (self)	Imputed data	0	36.75	36.47	0.29	(-1.10, 1.68)	0.68	0.02	0	36.76	36.97	-0.64	(-1.82, 0.54)	0.29	0.06
QoL-AD (proxy)	Complete case	11	31.2	31.7	-0.5	(-1.72, 0.72)	0.2	0.04	32	30.98	31.81	-0.84	(-1.9, 0.22)	0.46	0.09
QoL-AD (proxy)	Imputed data	0	31.22	31.46	-0.24	(-1.30, 0.82)	0.66	0.02	0	30.88	31.54	-0.7	(-1.74, 0.34)	0.18	0.07
AMIF	Complete case	22	49.75	49.18	0.58	(-3.32, 4.48)	0.85	0.02	9	52.18	49.84	2.34	(-0.95, 5.63)	0.14	0.08
AMIF	Imputed data	0	46.19	47.98	-1.79	(-5.55, 1.97)	0.35	0.05	0	51.61	50.16	1.45	(-1.29, 4.19)	0.3	0.06
AMIM	Complete case	22	12.56	12.24	0.16	(-1.39, 1.71)	0.49	0.01	9	11.31	11.24	0.59	(-0.8, 1.98)	0.24	0.05
AMIM	Imputed data	0	12.4	12.61	-0.36	(-1.71, 0.99)	0.6	0.03	0	10.72	11.07	0.17	(-0.95, 1.29)	0.77	0.02
QCPR (patient)	Complete case	52	57.66	56.99	0.67	(-1.00, 2.34)	0.65	0.05	48	57.32	56.84	0.48	(-1.46, 2.42)	0.84	0.03
QCPR (patient)	Imputed data	0	57.37	56.74	0.64	(-0.77, 2.05)	0.37	0.05	0	56.83	56.52	0.31	(-1.12, 1.74)	0.67	0.02
BADLS	Complete case	10	18.22	18.96	-0.74	(-2.50, 1.02)	0.2	0.04	28	17.17	16.65	0.52	(-0.83, 1.87)	0.42	0.04
BADLS	Imputed data	0	18.77	19.9	-1.13	(-2.50, 0.24)	0.11	0.09	0	17.32	16.84	0.48	(-0.83, 1.79)	0.47	0.04
RAID	Complete case	47	7.61	7.3	0.32	(-1.29, 1.93)	0.58	0.02	74	8.5	7.28	1.22	(-0.37, 2.81)	0.22	0.09
RAID	Imputed data	0	8.18	7.74	0.44	(-0.99, 1.87)	0.54	0.03	0	8.69	8.03	0.66	(-0.56, 1.88)	0.29	0.06
CSDD	Complete case	69	6.83	6.71	0.12	(-1.19, 1.43)	0.71	0.01	68	7.54	7.52	0.02	(-1.27, 1.31)	0.36	0.00
CSDD	Imputed data	0	7.31	6.94	0.38	(-0.68, 1.44)	0.48	0.04	0	7.78	8.07	-0.29	(-1.47, 0.89)	0.63	0.03
EQ-5D	Complete case	39	0.804	0.806	-0.001	(-0.06, 0.06)	0.72	0.00	48	0.764	0.756	0.008	(-0.04, 0.06)	0.95	0.02
EQ-5D	Imputed data	0	0.772	0.769	0.004	(-0.05, 0.06)	0.88	0.01	0	0.75	0.736	0.014	(-0.03, 0.06)	0.54	0.03
EQ-5D VAS	Complete case	41	70.55	70.96	-0.41	(-4.98, 4.16)	0.98	0.01	26	72.97	72.54	0.43	(-4.22, 5.08)	0.48	0.01
EQ-5D VAS	Imputed data	0	70.64	70.37	0.27	(-3.61, 4.15)	0.89	0.01	0	72.31	71.52	0.79	(-3.05, 4.63)	0.69	0.02
EQ-5D (proxy)	Complete case	17	0.588	0.626	-0.038	(-0.10, 0.03)	0.37	0.06	32	0.594	0.575	0.018	(-0.04, 0.07)	0.81	0.04
EQ-5D (proxy)	Imputed data	0	0.575	0.596	-0.021	(-0.07, 0.03)	0.44	0.04	0	0.584	0.56	0.024	(-0.03, 0.07)	0.36	0.05
EQ-5D VAS (proxy)	Complete case	12	62.1	63.2	-1.11	(-5.44, 3.22)	0.59	0.03	29	57.12	59.58	-2.46	(-6.58, 1.66)	0.67	0.07
EQ-5D VAS (proxy)	Imputed data	0	60.55	62.43	-1.88	(-5.66, 1.9)	0.33	0.05	0	56.48	58.97	-2.49	(-6.08, 1.1)	0.17	0.07

**Table 4:** Primary and secondary end point results adjusted analysis models for carer measures. Adjusted for carer age, baseline outcome score, centre, carer gender, relationship (spousal/non-spousal).

Measure	Data	Primary end point (10 month follow up)							Secondary end point (3 month follow up)						
		Missing (n = 350)	Treatment Mean	Control Mean	Group difference Mean	95% CI of MD	P values	Effect Size	Missing (N = 395)	Treatment Mean	Control Mean	Group Difference Mean	95% CI of MD	P values	Effect Size
GHQ28 (log transform)	Complete case	31	3.06	2.97	0.09	(-0.03, 0.21)	0.35	0.08	38	2.99	2.97	0.02	(-0.08, 0.12)	0.86	0.02
GHQ28 (log transform)	Imputed data	0	3.08	3.01	0.07	(-0.05, 0.19)	0.24	0.06	0	3.01	2.99	0.02	(-0.1, 0.14)	0.74	0.02
HADS Anxiety	Complete case	9	6.48	5.47	1	(0.1, 1.9)	0.15	0.12	28	5.98	5.64	0.35	(-0.39, 1.09)	0.61	0.05
HADS Anxiety	Imputed data	0	6.58	5.99	0.59	(-0.19, 1.37)	0.14	0.08	0	6.15	5.96	0.18	(-0.43, 0.79)	0.56	0.03
HADS Depression	Complete case	9	4.77	4.53	0.25	(-0.51, 1.01)	0.58	0.03	28	4.53	4.42	0.11	(-0.5, 0.72)	0.83	0.02
HADS Depression	Imputed data	0	5.12	5.03	0.09	(-0.64, 0.82)	0.81	0.01	0	4.69	4.76	-0.08	(-0.63, 0.47)	0.78	0.01
RSS	Complete case	14	22.03	21.55	0.48	(-1.64, 2.6)	0.95	0.02	31	22.96	21.99	0.98	(-0.76, 2.72)	0.21	0.06
RSS	Imputed data	0	22.93	22.87	0.06	(-1.86, 1.98)	0.95	0.00	0	23.22	22.48	0.75	(-0.84, 2.34)	0.36	0.05
QCPR (carer)	Complete case	17	53.13	53.06	0.07	(-1.89, 2.03)	0.45	0.00	43	51.61	53.1	-1.49	(-3.1, 0.12)	0.24	0.10
QCPR (carer)	Imputed data	0	52.13	51.57	0.55	(-1.17, 2.27)	0.53	0.03	0	51.39	52.57	-1.18	(-2.71, 0.35)	0.13	0.08
EQ-5D	Complete case	12	0.733	0.796	-0.064	(-0.12, 0)	0.11	0.11	31	0.782	0.772	0.01	(-0.03, 0.05)	0.77	0.02
EQ-5D	Imputed data	0	0.713	0.758	-0.044	(-0.09, 0.01)	0.09	0.09	0	0.776	0.761	0.014	(-0.02, 0.05)	0.44	0.04
EQ-5D VAS	Complete case	10	72.68	71.72	0.97	(-3.4, 5.34)	0.82	0.02	24	72.4	73.36	-0.96	(-5.04, 3.12)	0.64	0.02
EQ-5D VAS	Imputed data	0	70.47	69.44	1.03	(-2.69, 4.75)	0.58	0.03	0	71.54	71.48	0.07	(-3.44, 3.58)	0.97	0.00

There were no significant differences on secondary outcome measures, at either ten or three-month end points, apart from the finding that carers of people with dementia allocated to the reminiscence intervention had significantly increased anxiety on the General Health Questionnaire-28 sub-scale at the ten month end-point (mean difference 1.25, s.e. 0.5;, p = 0.04, 5 of the 5 multiple imputations also significant). There was no evidence of this difference at the 3 month time point.

### Compliance analysis

Taking attendance at six or more of the twelve weekly sessions as an index of compliance, on the basis of clinical consensus, 70% of those allocated to the intervention received it as planned. This fell to 57% when considering those dyads who additionally attended 3 or more of the monthly maintenance sessions. The number of weekly sessions attended was associated with improved performance on the Autobiographical Memory Interview (Memory) scale at the three month time-point (p = .023).

For the person with dementia, there was some evidence that improvement in the ten month score on the Quality of the Carer-Patient Relationship scale was associated with the number of monthly sessions attended (p = .043, 1 of 5 multiple imputations significant). This effect applied to the QCPR warmth sub-scale, but not the absence of criticism sub-scale. Number of sessions attended was also linked to a significant improvement in self-reported quality of life (EQ-5D utility) at ten months for people with dementia in the intervention group (p = .012 with three out of five multiple imputations significant).

However, carers showed increased stress related to caregiving associated with more sessions attended. At 10 months the number of sessions attended was associated with worse scores on the Relatives‘ Stress Scale (, p = .005 with four out of five multiple imputations significant). In the intervention arm, carers who continued to attend the reminiscence groups regularly scored 23.36 (se 1.33) but those who did not attend the groups or attended infrequently scored only 18.70 (se 1.77). Carers in the control arm scored 21.57 (se 1.19).

### Economic Analyses

#### Micro-costing of reminiscence group therapy and maintenance

Table 5 summarises the direct costs for 19 of the 28 RYCT programmes that ran over the course of the trial. For nine programmes cost data were either incomplete or unavailable (not collected). The cost per 19 session programme ranged from £4,215 to £14,579 with a mean cost of £9,433 (sd £2,651). Approximately two-thirds of the mean total cost of running a programme was accounted for by staff related costs, of which the largest sub-category was for group facilitators. Differences in employment arrangements for staff (freelance, with a fixed fee per session v. employed staff with additional employment costs) accounted for much of the variation between centres. The second highest costs related to transport for participants with dementia and their carers, varying considerably between programmes as transport had to be tailored to individual circumstances. The costing of reminiscence materials and resources was problematic. Costs presented in this category reflect the direct costs incurred by programmes in purchasing materials and resources and do not include any attempt at costing items brought to sessions by individuals/organisations, or recycled from previous sessions. The mean cost per dyad for the provision of a 19 session Remembering Yesterday Caring Today programme was £964 (based on a mean of 9.79 dyads per programme).

**Table 5:** Base case costs for Remembering Yesterday Caring Today (RYCT) programme of 12 weekly joint reminiscence groups and 7 monthly maintenance sessions based on data from 19 waves of recruitment.

	n^a^	Mean cost for the provision of each 19 session programme (£)	sd	Minimum (£)	Maximum (£)	Mean cost per session for 19 session programme (£)
**Training related**^b^	19	299	242	0	797	16
**Group facilitators**^c^^,^ ^d^	19	4,931	1,531	2,795	7,511	260
**Supporting staff (salary)**^d^^,^ ^e^	19	906	1,028	0	3,163	48
**Travel costs (facilitators and staff)**	19	266	461	0	1,900	14
**Sub-total (staff related)**		**6,402**				**338**
**Venue**	19	378	486	0	1,846	20
**Participant and carer transport**	19	2,258	1,583	100	4,750	119
**Reminiscence materials, resources, etc**.	19	158	114	0	330	8
**Refreshments**	19	185	38	95	237	10
**Administration**	19	52	35	0	102	3
**Totals**		**9,433**				**498**

^a.^ Number of recruitment waves for which data were available.

^b.^ Includes fees for reminiscence consultant and, where applicable, venue hire, salaries, freelancer fees, travel and subsistence.

^c.^ Facilitators comprised of both freelancers and NHS or university employees.

^d.^ Salary costs based on NHS Agenda for Change pay scales 2010/2011 and Bangor University Pay Scales 2010. To preserve privacy, salaries were calculated using the spine point nearest to the middle of the relevant scale.

^e.^ Costs calculated for supporting staff in NHS or university employment whose normal duties included activities connected to the running of the sessions (some paid assistants together with administrative and clinical support). Assistants who were NHS or local authority employees released from their normal duties to gain additional experience and skills were not costed.

#### Cost of service use

The costs of service use are derived from using national unit costs [30, 31]. Unit costs for each health and social care service were multiplied by the frequencies recorded in the Client Service Receipt Inventory completed by participants with dementia and carers. The analysis has been restricted to cases where full cost data could be calculated (i.e. where health and social care service use data had been obtained at both 3 and 10 month follow-ups). The price year used was 2010. Given that follow up was for less than 12 months discounting was not applied to either costs or outcomes.

Table 6 summarises the mean total costs of health and social care service use for the intervention and control groups for participants with dementia and carers over 10 months. While the mean total costs for participants with dementia in the intervention group were 13.5% (£580) higher overall than for the control group, this was not statistically significant. Most of the difference is accounted for by the higher mean cost of day care for the intervention group, which is 80% higher than the mean cost for the control group. This higher mean cost reflected the increased frequency of day service use (though not statistically significant overall) for participants with dementia in the intervention group. For carers, mean costs for the intervention group were 12.7% higher overall than the control group over the 10 month period (£171), although again this was not statistically significant.

**Table 6:** Summary of health and social service costs to the intervention and control groups over 10 months.

Service	Reminiscence (n = 196) Mean total costs (£)	SD	Control (n = 140) Mean total costs (£)	SD	Difference in mean total costs (£)	Asympt Sig^a^
**Participants with dementia**						
Community care	1,072	1,809	1,170	1,983	-98	0.674
Day care	1,098	4,451	610	1,415	488	0.230
Hospital use	2,719	7,106	2,529	8,087	190	0.801
Total (participant with dementia)	**4,889**	8,806	**4,309**	8,729	**580**	0.471
**Carers**						
Community care	258	339	283	449	-25	0.505
Day care	7	77	34	307	-27	0.400
Hospital use	1,266	3,752	1,043	3,622	223	0.694
Total (carer)	**1,531**	4,647	**1,360**	1,459	**171**	0.800
Grand total	**6,419**		**5,667**		**751**	

^a^: Asymptotic significances for Mann-Whitney U Test

#### Cost-effectiveness analysis

The incremental cost-effectiveness for the QoL-AD was £2,586 (i.e. the mean cost of a one point change on the scale reflecting an improvement in quality of life) (Table 7). It should be noted that the 95% confidence intervals for this estimate were extremely broad, and as they include zero, the ratio is not statistically significant. In view of the lack of clinical effectiveness, and the higher costs associated with the intervention group, the generation of cost-effectiveness acceptability curves was not appropriate.

**Table 7:** Summary of results of the cost-effectiveness analysis for participants with dementia using QoL-AD as a measure of effectiveness.

	Intervention (n = 196)	Control (n = 140)	Difference
**Mean total cost in £ (SD)**	5853 (8806)	4309 (8729)	1544
**Mean QoL-AD score (SD)**	37.013 (4.768)	36.416 (4.692)	0.597
**Incremental Cost-Effectiveness Ratio (£) (95% CI)**			2586 (-20280; 24340)

#### Cost-utility analysis

Cost-utility analysis was undertaken separately for participants with dementia and their carers using the total cost of health and social care services and QALYs generated from the self-completed EQ-5D. Cases included all those for whom complete cost data were available (n = 336).

While a full cost-utility analysis had been planned as part of the economic evaluation of the REMCARE trial, the results showed that generating cost-effectiveness acceptability curves would not be meaningful. The mean costs of health and social care service use were higher for the intervention group (Table 6) and the costs of the joint reminiscence and maintenance sessions only applied to the intervention arm, giving a higher overall mean cost for the intervention group compared to the control group. Coupled with the lack of any statistically significant effect from the self-reported EQ-5D, this indicated that the intervention could not be cost-effective. Results shown in Table 8 confirm that the mean difference in QALYs between intervention and control arms for both participants with dementia and carers were negligible. Given that these would generate meaninglessly high incremental cost per QALY figures, they have not been calculated.

**Table 8:** Summary of results of the cost-utility analysis.

	Person with dementia			Carer		
	Reminiscence (n = 196)	Control (n = 140)	Difference	Reminiscence (n = 196)	Control (n = 140)	Difference
**Mean total cost in £ (SD)**	5853 (8806)	4309 (8729)	1544	2495 (3866)	1359 (3743)	1136
**Mean QALYs (SD)**	0.644 (0.141)	0.643 (0.150)	0.001	0.632 (0.175)	0.633 (0.179)	-0.000

## Discussion

This trial does not provide support for the effectiveness of joint reminiscence groups for people with dementia and their carers in relation to intention to treat analyses of primary or secondary outcomes. At 10 months, carers in the reminiscence groups reported higher anxiety on the GHQ sub-scale whilst those allocated to usual treatment showed a reduced level of anxiety at this point. The compliance analysis suggests there are some benefits in terms of autobiographical memory, relationship quality and quality of life (EQ-5D) for people with dementia who attend sessions as planned. This must however be viewed in the context of potential raised stress in their carers and the lack of improvement on the dementia-specific quality of life measure (QoL-AD). The primary end point effectiveness results of this trial mean that the intervention could not be cost-effective. There were few statistically significant differences in the frequency and total cost of NHS, social care and voluntary sector service use between groups. The intervention group reported using more local authority and NHS day care services and day care hospital services than the control group over the ten month study period. It is not clear why this should be the case; it is possible that increased social contact through attending joint reminiscence groups may increase service uptake through improved knowledge of local service provision and availability.

This is by far the largest rigorous trial of any reminiscence intervention for people with dementia in the world literature and reports the first economic evaluation alongside a trial of joint reminiscence groups. Group facilitators received training from the originator of the approach and followed a detailed manual. The proportion of participants randomized to the intervention and receiving it as planned over the ten month period was less than 60%, with 11% attending no group sessions at all and a quarter attending three or fewer sessions.

The current study systematically collected only a limited amount of process data, and reasons for non-attendance are not clear. Attrition was greater in the treatment as usual group. The compliance findings may reflect a 'survivor' effect, with those able to continue having higher scores on certain measures, unrelated to the intervention. However, the analyses controlled for baseline levels of each measure, as well as key demographic factors.

One of the challenges of research in dementia care is to balance the outcomes for people with dementia and their carers, especially where these seem to potentially conflict. The current study used a limited range of carer outcome measures, which tended to focus on negative outcomes, rather than positive well-being. It is possible there may have been some benefits to carers which were not measured.

The lack of positive results from this study is unexpected and contrasts with the findings from our pilot trial platform [32] where 12 weeks of joint reminiscence groups were associated with significant benefits to both autobiographical memory and to carer depression; the control group showed a decline in these outcomes over the three months of the study, whereas those receiving reminiscence maintained their baseline levels. Similarly, the Cochrane review [7] of reminiscence for dementia also found significant improvements in cognition and mood after 4–6 weeks and reduced carer stress. The results are also not consistent with systematic reviews [5] suggesting that involving people with dementia and carers together in an intervention leads to better outcomes for family care-givers.

Three aspects are important to consider further, and are informed both by feedback which was collected systematically as part of the programme, and by a qualitative study involving in-depth interviews with 18 family carers who had attended RYCT groups in a subsequent study [33]. Firstly, the lack of positive findings stands in contrast to the reported enjoyment and benefits reported by participants and facilitators. Several groups chose to continue beyond the funded ten-month period. Of 215 participants responding to a feedback survey at the end of the weekly groups, all but 3 said that they would recommend the programme to a friend. There is an argument that interventions for people with dementia should be evaluated in relation to their immediate, within-session effects, rather than focusing on longer-term changes [34]. Secondly, despite signing up and consenting to a study involving reminiscence groups, many people did not take up the groups, or maintain attendance. For some, health problems or change of circumstances limited attendance, but some carers expressed discomfort with groups or expressed doubts about the potential benefits, and there were examples of people with dementia enjoying the groups but being withdrawn by their carer who was less enthusiastic. Outside the research context, joining such a group would be more a matter of personal preference with perhaps scope for the person with dementia to attend alone. It appears that other modalities for enhancing relationships between people with dementia and their carers need to be explored. Thirdly, this study has highlighted increased anxiety in carers, with higher stress reported by carers attending a greater number of group sessions. Examples of similar negative effects have been reported in other studies where the carer has participated in a therapeutic approach with the person with dementia [35,36]. The increased use of day care in the intervention group may be relevant, with one study suggesting that day care may be associated with lower carer mood [37]. Documented negative aspects of the intervention, from the perspectives of carers include exposure to people with more advanced dementia offering an ‘unwelcome vision of the future’; disappointment when the person responds well in the group, and then returns to their usual condition later; lack of respite and lack of attention to the carer’s needs, and feeling guilty at not being able to implement new skills learned in the group [33]. These results should encourage reappraisal of the move towards encouraging joint interventions which reflect the current emphasis on ‘relationship-centred care’ [38]. Many people did engage with the intervention, and clearly enjoyed the activities and social aspects. The expectation that interventions in dementia care should always have a sustained benefit outside the immediate context may also need to be re-considered, so that clear expectations for participants and practitioners may be established. Reminiscence may be one example of a leisure activity, enjoyed by many, that can contribute to a rich and varied lifestyle for people with mild to moderate dementia.

## Transparency

The trial protocol is available at http://www.biomedcentral.com/content/pdf/1745-6215-10-64.pdf and the full study report at http://www.journalslibrary.nihr.ac.uk/hta/volume-16/issue-48. All authors had full access to all of the data (including statistical reports and tables) in the study and can take responsibility for the integrity of the data and the accuracy of the data analysis. The lead author (RTW) (the manuscript's guarantor) affirms that the manuscript is an honest, accurate, and transparent account of the study being reported; that no important aspects of the study have been omitted; and that there are no discrepancies from the study as planned.

## Supporting Information

S1 CONSORT Checklist Consort checklist for the REMCARE study. (PDF)

S1 Protocol REMCARE protocol approved by Ethics Committee. (PDF)
