# Supplementary material for: REMCARE: Pragmatic Multi-Centre Randomised Trial of Reminiscence Groups for People with Dementia and their Family Carers: Effectiveness and Economic Analysis
Source: PLoS One. 2016 Apr 19;11(4):e0152843. doi: 10.1371/journal.pone.0152843 (PMC4836678; doi:10.1371/journal.pone.0152843)
Supplement: S1 Protocol — (PDF) [file pone.0152843.s002.pdf]

REMCARE

Reminiscence groups for people with dementia and their family care-givers: pragmatic 8-centre trial of joint reminiscence and maintenance v usual treatment

REMCARE Protocol

Version 1.2: 14th September 2007

ISRCTN042430123

Project title
Reminiscence groups for people with dementia and their family care-givers: pragmatic 8-centre trial of joint reminiscence and maintenance v usual treatment (REMCARE)

Planned investigation

Research objectives
1 To compare the effectiveness (in ameliorating the quality of life of people with dementia & the stress on their carers) of joint reminiscence groups with participants & carers followed by reminiscence-based maintenance with that of 'usual treatment'.

2 To compare the incremental cost-effectiveness (in ameliorating the quality of life of people with dementia & the stress on their carers) of joint reminiscence groups with participants & carers followed by reminiscence-based maintenance with that of 'usual treatment'.

Existing research
The development and evaluation of therapeutic interventions intended to benefit people with dementia and their family care-givers is the subject of much research interest at present. In view of the large and growing numbers of people with dementia, and the costs associated with meeting needs for care, there are clear advantages for health and social care services if people with dementia can be supported in the community for an extended period, with less intensive support. However, there is consensus that this must not be at the cost of additional burden to family care-givers (1).

Most attention has been given to pharmacological interventions, but there is increasing recognition that psychosocial interventions may have comparable value (2, 42), and may be preferable in some contexts, e.g. where medication may have negative side-effects (3, 42). A number of systematic reviews of psychosocial interventions are now available (e.g. 1, 4, 5), as well as a number of Cochrane reviews of specific approaches (e.g. 6, 7).

In practice, in the UK, Reminiscence Therapy appears to be the most well-known therapeutic approach to working with people with dementia. For example, over half of care homes in Wales claim to offer this approach to their residents (8). Reminiscence work with people with dementia has an extensive history (9, 10), involving enjoyable activities that promote communication and well-being. One factor in its popularity is that it works with early memories, which are often relatively intact for people with dementia, thus drawing on the person's preserved abilities, rather than emphasising the person's impairments. However, its popularity has not led to a corresponding body of evidence on its effects. The existing research literature has been brought together in our revised Cochrane review on reminiscence therapy for people with dementia (11). Only four randomised controlled trials (RCTs) suitable for analysis were identified. Each examined different types of reminiscence work; all were small or of poor quality. Taking the results from the studies together, some significant results were obtained in relation to cognition and mood 4-6 weeks after the treatment, and reduced care-giver stress where the care-giver participated with the person with dementia in a reminiscence group. However, the review concludes that 'in view of the limitations of the studies reviewed, there is an urgent need for more quality research in the field'. This dearth of evidence is reflected in the NICE-SCIE Guideline on the management and treatment of dementia (42), which found insufficient evidence to recommend that reminiscence should be routinely offered to people with dementia, although its potential impact on mood of the person with dementia was highlighted.

In order to take research forward, there is a need to specify clearly the exact nature of the reminiscence work to be undertaken and its aims. Typically, a group approach has been used, with 'memory triggers' (photographs, recordings, artefacts etc.) used to promote personal and shared memories. A recent development has been to involve family care-givers in the groups alongside their relatives with dementia. Descriptive evaluations suggest that this joint approach (described as 'Remembering Yesterday Caring Today' - RYCT) may improve the relationship between care-giver and person with dementia, benefiting both (12). As it is the breakdown of this care-giving relationship that increases the likelihood of the person with dementia being placed in institutional care, this effect could have far-reaching implications for families, society and public spending. Our group have reported a very small pilot study evaluating this joint reminiscence approach (7 patient-carer dyads in the treatment group; 4 in the waiting-list control group), which showed some trends in improved

quality of life for patients and reduced stress for care-givers (13). In the next section, we shall present results from a larger trial platform, funded by the MRC, that has recently been completed, which developed this pilot work further.

The justification for evaluating the joint reminiscence approach specifically comes from this promising pilot data and the great interest in this approach in the field of reminiscence work (9). More generally, a recent meta-analysis (1) on interventions with family care-givers of people with dementia suggested that joint approaches may be more effective in improving care-giver outcomes than approaches targeted only at the care-giver. The previous tradition in dementia care of interventions for people with dementia and their care-givers separate from each other is being questioned. For example, in many areas of the UK, Alzheimer Café sessions have been established, with an agenda including education as well as social contact, attended by both people with dementia and their care-givers. The emphasis has shifted from 'person-centred care' to 'relationship-centred care', with recognition of the central importance of the relationship between person with dementia and care-giver to the well-being of both. Although a joint focus on people with dementia and their care-givers is not applicable to all people with dementia, the proportion of people with dementia without an identifiable care-giver has been reported to be as low as 6% (14), with such people being much more likely to enter care homes.

Reference methods
It is proposed to carry out a pragmatic randomised controlled trial of joint reminiscence groups v usual treatment.

<u>Trial platform</u>
The applicants have recently completed (31st May 2006) a pilot study comparing these joint reminiscence groups with usual treatment as part of a trial platform funded by the Medical Research Council (MRC), which also refined outcome measures and prepared a detailed treatment manual. The trial platform also included an additional condition where people with dementia attended reminiscence groups without their carers.

Methods
Three centres participated in the trial (Bangor, Bradford and UCL). Across the centres, three joint groups and two reminiscence alone groups were run. Participating dyads were randomised to either the joint reminiscence condition or to an active control condition (reminiscence alone) or a passive control condition (treatment as usual), depending on the centre. In the Bradford centre, the Zelen randomisation method (15) was trialled; participants initially agreed to complete the assessment procedures at each time-point; if randomised to an active intervention, further informed consent was then sought.

Participants were recruited from local NHS services, including Memory Clinics, and from voluntary agencies, such as the Alzheimer's Society. Inclusion criteria were a diagnosis of mild to moderate dementia and the absence of severe agitation and communication problems. All participants were required to have a family care-giver able and willing to attend reminiscence sessions with the person with dementia. 65 participant dyads entered the trial and provided baseline data (see Appendix 1: CONSORT diagram for trial platform). 57 went on to receive the intervention they were randomised to (7 being lost through stage 2 Zelen refusals). The post-treatment assessment was completed by 50 dyads; a three-month follow-up assessment was completed by 45 dyads (10 treatment as usual, 24 joint reminiscence, 11 reminiscence alone).The median age of the people with dementia was 78 years; that of the care-givers was 72 years. The average Mini-Mental State Examination (16) score was 19.3 (sd 5.0) (moderate dementia: 12-20; mild dementia 21-26).

Primary outcome measures were: QoL-AD (17), a quality of life measure completed with the person with dementia in a structured interview, which is also completed on a proxy basis by the care-giver; Relatives' Stress Scale (18), a self-report measure of the direct impact of care-giving. Secondary outcome measures included: a measure of autobiographical memory (the type of personal memory over the life-span that should be influenced by reminiscence work), adapted for the project to include more items and better coverage of the life-span; measures of care-giver distress and depression (the General Health Questionnaire (GHQ-28) (19) and the Geriatric Depression Scale (GDS-15) (20); measures of the quality of relationship between the person with dementia and care-giver (Quality of Care-giver Patient Relationship – QCPR) (21), ratings of video-taped interactions between person

with dementia and care-giver in two structured situations (22).

Results
All analyses reported were undertaken using analysis of covariance on post-treatment (or follow-up scores), with baseline scores as the covariate. For most of the measures in this small sample, differences between joint reminiscence and reminiscence alone were small. For the primary outcome measures, comparing either type of reminiscence with treatment as usual, the differences were not statistically significant; the effect sizes for QoL-AD, rated by the person with dementia were small at post-treatment (0.17) and at 3 month follow-up (0.40); the initial rating for the care-giver rating of the quality of life for the person with dementia (a secondary outcome) was slightly higher (0.50), but the effect size at three-months was similar (0.33). On the primary outcome for care-givers, the Relatives' Stress Scale, effect sizes were small to moderate (0.36 and 0.31).

On secondary outcome measures, people with dementia in the joint reminiscence group had significantly better autobiographical memory at post-treatment than those receiving treatment as usual (effect size 0.61; p=0.007), but this was not maintained at follow-up. Care-givers involved in the joint reminiscence group reported less depression at post-treatment than those in the treatment as usual condition, a difference that was maintained at follow-up (effect size 0.57; p=0.013 and effect size 0.42; p=0.024 respectively). These findings were also clear when treatment as usual was compared with either type of reminiscence, with reminiscence work associated with better autobiographical memory at post-treatment, but not follow-up, and the reminiscence conditions also associated with reduced care-giver depression and distress (on GHQ) at post-treatment and (on GDS and GHQ) at follow-up. Effect sizes for all these comparisons were in the range 0.48 to 0.6, except for autobiographical memory at follow-up, which was 0.13. The details of the comparisons between any form of reminiscence and treatment as usual are shown in Appendix 1.

Implications of *trial platform for full trial proposal*
a) The Zelen method of randomisation led to several refusals to accept experimental interventions, thus weakening the effect of those interventions as Zelen analyses by 'intention to treat'; as there was no evidence that it otherwise assisted recruitment and retention in this field, we shall not use it in the proposed trial.
b) Though the trial platform necessarily generated wide confidence intervals, the difference in effects between joint reminiscence and reminiscence alone appear to be small, as one might have predicted a priori from the similar resources allocated to each. Indeed, reminiscence alone may have beneficial effects for care-givers also. This may be because of the brief respite afforded to the care-giver, or from the benefits they perceive the person with dementia receiving.
c) Although the further comparison of joint reminiscence and individual reminiscence would be of interest in providing a test of the additional effects of joint working, and of relationship-centred care, we are persuaded that the size and complexity of trial that would be required, given the probable small effect size for any difference between them, would not be feasible. Accordingly, we are now proposing to focus on joint reminiscence groups.
f) Participants in the joint reminiscence groups requested monthly reunion meetings following the end of the 12 weekly sessions. They wished these to continue to have a reminiscence focus in addition to social contact. These maintenance sessions over the follow-up period have been built into the current proposal.
g) It proved entirely feasible in the trial platform to recruit to two arms over a 6 month period in each centre.
h) The trial platform thoroughly tested the outcome measures for this population, which appear valid, reliable, responsive, relevant and acceptable.
i) The treatment manual for the joint intervention has been refined and tested. The training procedure for new group facilitators has been developed and adjusted. A simple treatment adherence schedule has been developed, which can be completed directly by a trained observer.

<u>Recruitment and randomisation</u>
As in the trial platform, recruitment will be through mental health services for older people in each area (especially Memory Clinics, Community Mental Health Teams for Older People and associated professionals including psychiatrists, occupational therapists and Admiral Nurses), associated day services and through relevant local voluntary sector agencies such as the Alzheimer's Society. Recruitment will be in three waves, offering the opportunity to focus on different geographical areas

within the remit of each centre for each group. In each centre, there will be a six month period between one group commencing and the next; recruitment in each centre to the Trial Platform was achieved well within this period.

As in the trial platform, which had approval from the relevant LRECs, the project would be briefly outlined to the potential participants by a member of the clinical team or Alzheimer's Society worker, and permission to contact with a member of the research team obtained. The research worker would then arrange to meet the potential participants and offer full details, respond to questions etc. and, where the participants were willing to join the study, undertake the process of consent. The NHS service costs associated with this proposal include an amount for the initial explanation of the project by the clinical team member and obtaining of the potential participants' permission to introduce them to the research team. In the current application, this important process will be facilitated by the joint appointments of several of the research team with local NHS Memory Clinics and other services (Woods, Orrell, Moniz-Cook, Keady). In addition, in the Hull centre, there is an existing protocol where all Memory Clinic attendees are given the opportunity to give consent at the outset to be approached regarding future research projects in which the service is participating. NEURODEM Cymru has funding to introduce a similar 'opt-in to research' system to Memory Clinics and other services in Wales by the commencement of the project groups, which will similarly ensure that only those with some interest are approached. In each case, those who have opted in are under no obligation to participate in any particular project.

The local researcher who will not take part in any follow-up assessments will contact the remote randomisation service of the North Wales Organisation for Randomised Trials in Health (NWORTH) when they have 24 dyads ready for randomisation. NWORTH is a trials unit recognised & funded by the Clinical Research Collaboration Cymru specifically for HTA trials. The same researcher will make arrangements for the 12 dyads randomised to the intervention group to attend group sessions, and will liaise with the group facilitators.

<u>Other biases</u>
Trials of psychosocial interventions cannot be blind to therapists or participants because they are aware of which, if any, treatment they are delivering or receiving. In contrast, researchers who assess participants after randomisation should not know to which arm they belong. In particular post-treatment and follow-up assessors will not attend any of the group or maintenance sessions, and will not have access to attendance lists etc.

However, our experience in the trial platform (shared by similar projects) is that participants may occasionally and inadvertently inform researchers of the treatment they are receiving. We aim to reduce this effect by explicit reminders to participants before the assessment visit, and by the use of self-report measures wherever feasible. We shall also ask all assessors to record their impression of the arm to which each participant belongs, and their confidence in that prediction. This will enable us to test whether inadvertent loss of blinding leads to bias, and to adjust for any bias detected..

Centres
The proposal is based on the involvement of 8 centres. These are as follows:

1) Bangor; this centre will recruit from the 3 NHS Trusts in North Wales, running groups in each area across the 3 waves. Support in recruitment will be secured from NEURODEM Cymru, the Wales Dementias & Neurodegenerative Diseases Research Network, for which Professor Woods is the academic lead. The 4 Memory Clinics in North Wales already collaborate on research projects.
2) South Wales (Newport), again with support from NEURODEM Cymru. This service has a number of sectors covering distinct geographical areas.
3) London – Essex; this centre will recruit primarily from the North East London Mental Health Trust, covering 4 London boroughs with a population of 120,000 older people, and 3 Memory Clinics.
4) London – South; this centre will recruit from the Memorial Hospital, Woolwich and associated services, which participated in the trial platform, and where RYCT groups have been running since

1998.
5) Hull; this centre will recruit from Humber Mental Health Teaching NHS Trust and adjoining areas. Their Memory Clinics work closely with the Alzheimer's Society in Hull, and cover a population of 80,000 older people.
6) Bradford: this centre will recruit from Memory Clinics and Alzheimer's Society groups in Bradford.
7 & 8) Manchester; this double-centre will recruit from the large populations covered by the Bolton, Salford and Trafford Mental Health Trust, including specialist Admiral Nurses in Bolton and other well-developed services.

Adoption of the project by DeNDRoN UK has been discussed with the relevant Clinical Studies Groups and the project appears to meet the various criteria specified. In Wales, the project would be adopted by CRC Cymru through NEURODEM Cymru. Support for recruitment will be sought from the respective Research Professional Networks, following adoption. The Manchester and North London centres fall within the areas of DeNDRoN Local Research Networks, and the two Wales centres will receive support from NEURODEM Cymru. Bradford, Hull and South London are located outside areas covered by a thematic local research network, but if required, could access support from the Comprehensive Research Networks which are planned to be in place by the time the project commences.

Planned interventions

Joint reminiscence groups (JRGs)

This approach is known as 'Remembering Yesterday Caring Today' (RYCT). It places emphasis on active, as well as passive forms of reminiscence, involving both care-givers and the person with dementia. Couples will attend 12 two hour sessions, held, where possible, in a social as opposed to a clinic-based setting. Each session is structured around a different theme for example; childhood, schooldays, working life, marriage, and holidays and journeys. Couples are encouraged to contribute with materials brought from home. Each session involves a blend of large and small group work. Typical activities include art, cooking, physical re-enactment of memories, singing and verbal reminiscence. The emphasis is firmly placed on the inclusion of the person with dementia. In the joint reminiscence groups care-givers are guided by facilitators and volunteers into allowing time for the person with dementia to respond and to value the contributions of the person with dementia.

There is a maximum limit of 12 couples to two trained facilitators in each group, together with a number of trained volunteers. Our previous experience suggested that ideally volunteers should be a mixture of ages drawn from voluntary sector (Alzheimer's Society, Age Concern), psychology graduates and former carers with an understanding of working with older people. The training programme for facilitators and volunteers is set out in the RYCT manual (developed during the MRC trial platform). Training involves acquiring skills in listening, group dynamics, interpretation of behaviours and learning methods to maximise inclusion of carers and people with dementia. Two half day training sessions take place before the group commences. After each session time is set aside for the facilitators and volunteers to prepare session notes and to complete attendance and evaluation forms. Evaluation forms from care-givers and people with dementia are collected at the end of the first session and at the end of the 12 week programme. The RYCT manual provides facilitators and volunteers with a recommended blend of activities for each session, based around the core principles of RYCT.

The availability of volunteers means that if, for any reason, carers are not able to attend all the group sessions, the person with dementia can still be involved and engaged in the group sessions. Maintenance sessions are held monthly, and follow a similar pattern – re-visiting some topics and introducing some new ones such as considering a particular decade, e.g. the 1950s, with the aid of relevant music and video clips.

Treatment as usual

The services and interventions available to people with dementia and family care-givers randomised to receive usual treatment will naturally vary between and within centres and may change over time. In general, the interventions offered to this group will also be available to those in the active treatment groups, so that we will be examining the *additional* effects of reminiscence work. The only exception to this would be where the active treatment is scheduled at the same time as an alternative

intervention. Our approach to costing the services and interventions received should allow us to monitor whether the usual treatment group is receiving alternative interventions in this way. Changes and developments in the availability of medications for Alzheimer's and other dementias will affect both groups equally, and will be recorded as part of the costing information collected.

It is entirely feasible that participants in the usual treatment group may be involved in some form of reminiscence work during the 10 months of the study period. It is a popular approach in day-care centres; reminiscence materials are widely available. However, it is very unlikely that, in our experience, such a structured approach to reminiscence work will be offered in any of the centres, or that it will be offered jointly to carers. It is this systematic group-based approach, rather than a general exhortation to reminisce to improve communication, that is the concern of this evaluation.

Drop-out rates
It is anticipated that some participants will not complete the full number of treatment sessions; in the trial platform, ill health was usually cited as the reason; 12% of participating dyads were lost between beginning the active treatment and the post-treatment evaluation. Our sample size calculations allow for this attrition. Wherever possible, the dyad will be encouraged to continue with the assessment measures, allowing them to be included fully in an intention to treat analysis, irrespective of number of group sessions attended. .

Adherence to treatment protocol
In order to check on the parity of treatment across different centres, in the Trial Platform we have developed a simple adherence to treatment schedule; sections of a small sample (around a quarter) of group sessions will be observed by a trained rater and rated on specific aspects of RYCT; for example aspects of communication, session structure and thematic content. These observations will be fed back to facilitators and will support the supervision process.

Recruitment and training of facilitators
The reminiscence groups require skilled facilitators to lead them. The presence of family care-givers, and the requirement to ensure that their concerns do not dominate the group, adds a further dimension. One of the aims of our trial platform was to ensure that new facilitators could be recruited and trained to carry out the approach in line with the principles established by its originators, Pam Schweitzer and Age Exchange. Accordingly, whilst Pam Schweitzer led one of the reminiscence groups in the trial platform, the remaining four were led by facilitators who received initial training from Pam Schweitzer, together with the opportunity to discuss issues as they arose once the groups were underway. These facilitators included an experienced community mental health nurse, an occupational therapist and a health care assistant, with several years experience in a very active dementia day-care service, and community arts workers.

In the proposed trial, facilitators will be identified in collaboration with each centre, and training and supervision provided. We anticipate that the majority of facilitators will have a mental health nursing or occupational therapy or clinical psychology background, but large group facilitation skills, warmth, energy and enthusiasm are as important as any particular professional qualification.. The use of two facilitators for each group, and the inclusion of volunteers, enables effective de-briefing and learning to occur at the end of each session. Group facilitators will participate in monthly supervision sessions, with a supervision team including the project consultant Pam Schweitzer, and arrangements for more immediate access to supervision will also be made. The training programme for volunteers is set out in the RYCT manual (developed during the MRC trial platform). Training covers skills in listening, group dynamics, interpretation of behaviours and learning methods to maximise inclusion of carers and people with dementia. Two half day training sessions take place before the group commences. After each session time is set aside for the facilitators and volunteers to prepare session notes and to complete attendance and evaluation forms. The RYCT manual provides facilitators and volunteers with a recommended blend of activities for each session, based around the core principles of RYCT.

Planned inclusion criteria
(1) Participants with dementia will meet the DSM-IV (24) criteria for dementia. All types of dementia will be included, including Alzheimer's, vascular dementia, Dementia of Lewy Body type and mixed dementias.

(2) Participants with dementia will be in the mild to moderate stage of dementia (Clinical Dementia Rating: (25)).

(3) Participants with dementia will have some ability to communicate and understand communication: a score of 1 or 0 on the relevant items of the Clifton Assessment Procedures for the Elderly – Behaviour Rating Scale (26).

(4) Participants with dementia will be living in the community at the time of the baseline assessment, and will have a relative or other care-giver who maintains regular contact, can act as an informant, and would be willing and able to participate in the intervention with the person with dementia.

Planned exclusion criteria
(1) Participants will not have a major physical illness or sensory impairment or disability or high level of agitation which could affect participation.

Ethical arrangements
Risks and anticipated benefits for trial participants:
There appear to be no documented harmful side-effects from participating in reminiscence groups and no adverse reactions were apparent in the MRC trial platform. Some past memories can be unhappy, and even traumatic, but with a skilled and trained facilitator participants will share only those aspects they feel comfortable with, and if distressing memories were to surface, the person would be given additional support on a one-to-one basis.

Benefits are consistently reported by participants in the groups, including enjoyment, feelings of validation and self-worth. The desire of participants to continue meeting following the sessions provides an indication of the value placed on the benefits. Prospective participants will be fully informed of the potential risks and benefits of the project.

Consent:
Participants will be in the mild to moderate stages of dementia, and therefore would generally be expected to be competent to give informed consent for participation, provided that appropriate care is taken in explaining the research and sufficient time is allowed for them to reach a decision. In every case, the participant will have had at least 24 hours to consider the information provided. It is helpful for a family member or other supporter to be involved, and we would aim to ensure that this is done wherever possible. Informed consent will be sought separately from the family care-giver, in relation to their own participation. It will be made clear to both participants and family care-givers that no disadvantage will accrue if they choose not to participate.

In seeking consent, we will follow current guidance from the British Psychological Society on evaluation of capacity. In this context, consent has to be regarded as a continuing process rather than a one-off decision, and willingness to continue participating will be continually checked through discussion with participants during the assessments.

Where the participant's level of impairment increases, so that they are no longer able to provide informed consent, the provisions of the Mental Capacity Act will be followed, with the family care-giver as consultee. Where the person has themselves given informed consent initially, this provides a clear indication of the person's likely perspective on continuing at later time-points. The same procedure will apply where the person with dementia appears to lack capacity to consent initially, but meets the other criteria for the project. At any point where a participant with dementia becomes distressed by the assessments they will be discontinued.

Retention of trial documentation:
It is planned that anonymised data will be kept securely for a period of seven years following the completion of the trial, subject to discussion with relevant Ethics Committees.

Confidentiality
Only members of the research team will have access to the original data. Participants' personal details will be stored separately from the data, and will be kept in a separate file on a password protected computer at the University of Wales Bangor. Each participant will be assigned an identification code,

which will be used in all data storage files; these will not contain names or any other means of personal identification. All personal details will be deleted on completion of the study.

Proposed sample size

Our target sample size is 400 patients *completing data collection* for the trial after ten months, comprising 200 in JRGs and 200 receiving treatment as usual. In the trial platform intra-class correlation coefficients (ICCs) within randomised groups were negative (i.e. not significantly different from zero) for both the carer-specific GHQ-28 and the carer-rated QoL-AD, but close to 0.1 for the QoL-AD rated by the person with dementia. Using a 5% significance level, comparison of the 200 pairs completing JRGs with the 200 people with dementia receiving treatment as usual will yield 80% power of detecting a standardised difference of 0.28 in the GHQ or the carer-rated QoL-AD. In contrast the patient-rated QoL-AD is likely to suffer a 'variance inflation factor' of approximately 1.74 [viz. 1 + 0.1 x (average completed group size of 8.4 minus 1)], thus yielding a power of 80% of detecting a standardised difference of 0.38. Our trial platform, which had a sample size of 57 in 3 centres, suggests that these differences between 0.28 and 0.38 are plausible. In our judgement they also fall within the range of effects that are clinically important. Furthermore, because our trial platform was exploratory, and therefore more heterogeneous than the proposed definitive trial, ICCs and standard deviations are likely to fall. To achieve a sample size of approximately 400, we need to allow for 12% attrition between recruitment and the post-treatment assessment (estimated from our trial platform) and a further 18% over the following 7 months (estimated from a community study (27)). Hence we shall seek an initial sample size of 576, requiring 24 treatment groups initially comprising 12 dyads and another 288 randomised to usual treatment.

Statistical analysis

We shall analyse by intention to treat, in that all available data will be included, however methods of imputation such as LOCF are of limited utility in dementia, where the expectation is decline for the usual treatment group, and participants will be lost through death and illness. Hence our sample size calculations are based on the numbers estimated to be available at the study end-point, ten months after randomisation. Multi-level modelling will be used to address the issue of clustering within randomised groups. We shall also use analysis of covariance to adjust for baseline differences in outcome variables. Analyses will consider the evaluation ten months after randomisation as the primary end-point in evaluating whether the intervention has had a substantive effect on the person with dementia and/or care-giver. Secondary analyses will consider the effects immediately following the intensive phase of 12 weekly group sessions.

Proposed outcome measures

Primary outcome measures:

a) quality of life for the person with dementia, self-assessed by the *QoL-AD* (17), which has been shown to be reliable and valid for people with mild and moderate degrees of dementia (28), (29). The scale is completed in a structured interview with the person with dementia and covers 13 domains of life quality.

b) care-giver's mental health, evaluated using the 28 item, self-report *General Health Questionnaire* GHQ-28 (19) which has been widely used in care-giver research (30, 31); the Likert scoring system 0-1-2-3 will be used. The scale includes indicators of anxiety, depression, insomnia, social dysfunction and somatic symptoms. This is preferred as the primary care-giver outcome to the Relatives' Stress Scale in this study, in view of its more general focus and wide usage.

Secondary outcome measures:

a) Autobiographical memory, assessed using an extended version of the *Autobiographical Memory Interview (*32). The extended AMI assesses recall of the person with dementia's personal memories relating to both factual (semantic) information for example, names of schools or teachers and specific incidents. In the trial platform, we validated an additional section on middle-age to retirement, to give systematic coverage to the life-span of our participants.

b) Measure of relationship quality, self-completed by both person with dementia and carer: *Quality of the Care-giving Relationship*: QCPR (21). Originally developed in the Netherlands this scale comprises 14 items (with 5 point Likert scales) designed to assess the warmth of the relationship and the absence of conflict and criticism. In the trial platform, the QCPR had good internal consistency for carers α.85 and for people with dementia α.80 and concurrent validity with other measures of

relationship quality and carer stress.

c) Depression and anxiety for both people with dementia and carer (Cornell Scale & RAID for person with dementia; Hospital Anxiety & Depression Scale for carer);
Cornell Scale for Depression in Dementia (CSDD) (33): A 19-item interviewer administered measure, using information from interview with the person with dementia and their carer. Signs and symptoms are described to the carer as they appear on the scale. Where there is a discrepancy between the carer and clinician's ratings the carer is re-interviewed before the interviewer makes the final judgment.
RAID (34): An 18 item rating scale to measure anxiety in a person with dementia based on a structured interview with the carer and the person with dementia.
The *Hospital Anxiety & Depression Scale* (35) is a 14-item, self-report well-validated scale, which provides an index of both anxiety and depression, and is suitable for use with adults of all ages.

d) Stress specific to the care-giving situation - the *Relative's Stress Scale* (18): self-report scale for the care-giver, contains 15 items rated on a 5-point Likert scale.

e) Quality of life of person with dementia, rated by the care-giver, using the proxy version of the *QoL-AD* (17), identical in structure and content to the self-report version above.

f) Costs, using the validated *Client Services Receipt Inventory* (CSRI) (36). The CSRI has been used extensively in studies of mental health and dementia care (e.g. (37)) and comprehensively gathers data on accommodation, medication and services accepted. In this case, the data collected will reflect the previous 3 months (at baseline and post-treatment) and 7 months (at follow-up).

g) Quality of life of care giver and person with dementia will also be measured using *EQ-5D*. EQ-5D (49) is a standardised instrument for use as a measure of health outcome, applicable to a wide range of health conditions and treatments. It provides a simple descriptive profile and a single index value for health status. EQ-5D was originally designed to complement other instruments but is now increasingly used as a 'stand alone' measure. EQ-5D is designed for self-completion by respondents and can be used in face-to-face interviews. It is cognitively simple, taking only a few minutes to complete. Instructions to respondents are included in the questionnaire. We did not include the EQ-5D originally, in view of concerns that use of a generic quality of life measure such as EQ-5D might not be sufficiently sensitive for use as the primary outcome measure with people with dementia. Our team has previously used the EQ-5D to evaluate the concurrent validity of the QoL-AD (28), and the two scales showed moderate correlation (0.54), but rather less of the sample of people with mild to moderate dementia were able to complete it, even though it was administered in an interview. Care-givers will be asked to complete the measure from their own perspective and for the person with dementia. The self-report of the person with dementia will also be obtained wherever possible.

h) The Bristol Activities of Daily Living Scale (50), a 20 item scale, completed by the carer, rating the functional ability of the person with dementia

Health Economics Analysis
Our Approach
In this study, our principal chosen method of economic analysis is cost-effectiveness analysis. The study population offers an opportunity for us to conduct a secondary cost-utility analysis, and for transparency, we plan to set out all costs and effects for people with dementia and their carers in a cost-consequence analysis.

Cost data
This analysis takes a multi-sectoral public sector perspective spanning the NHS (dementia Services, primary and secondary care) and local government. The interventions received will be fully costed from the perspective of local dementia services to generate a total programme cost and cost per participant or per participant-carer pair.

We shall estimate the costs of dementia care through the validated Client Service Receipt Inventory (CSRI), completed with the family care-giver. The measurement of health service utilization is a routine part of the estimation of costs in economic evaluation. There is a growing literature on the reliability of patient recall as an alternative to accessing GP records, (e.g. 47) and our economic

protocol is consistent with that used by health economists who have conducted trials in this field previously (36, 37). GP and other provider records are not necessarily an entirely accurate source of service utilization and hence costing information. These formal records, though mainly computer based, are sometimes incomplete or not sufficiently linked between provider agencies e.g. primary and secondary NHS care, NHS and social services. We consider that the costs of collecting data from GPs and other care providers for the whole sample would not be justified in terms of adding accuracy or reliability to the utilization and costing information used in the planned evaluation. We propose to triangulate with GP notes for a sub-sample to enable the estimation of any systematic differences in reports. What is important is that control and intervention groups are treated identically in terms of costing, as it is the difference in costs and effects between groups that is of interest. The triangulation exercise will be conducted with 40 participants (20 in the intervention group and 20 in the control group) to compare self-reported visits to primary and secondary care with recorded visits on GP notes for the 10-month study period, to validate this approach. We will use National costs (38, 44).
Costs will include:

- Costs of running the joint reminiscence groups.
- Costs of reminiscence-based maintenance groups following the initial intervention.
- Direct costs of all primary and secondary health care services used by participants in the intervention and control arms of the study (home/surgery telephone contacts with GP and practice nurse, outpatient and inpatient attendances at secondary care, prescribing).
- Indirect costs associated with lost productivity and care-giver costs of attending group sessions.
- (No intangible costs to be included).

Effectiveness Data
Effectiveness will be evaluated in terms of the primary clinical outcomes: the specific quality of life measure QoL-AD and the GHQ-28 at the primary end-point..

Incremental Cost-effectiveness Analysis
The incremental cost-effectiveness ratio will indicate the change in costs and effectiveness of moving to joint reminiscence group therapy followed by reminiscence-based maintenance for the improvement of quality of life of people with dementia and amelioration of care-giver stress, as compared with no intervention. We will use bootstrap calculations for examining the uncertainty in the cost-effectiveness analysis, to provide an estimate of the probability distribution of the cost-effectiveness ratio, its confidence interval, or variance in the ratio. We will plot cost-effectiveness acceptability curves (CEACs), which have been widely adopted as a method to quantify and graphically represent uncertainty in economic evaluation studies of health-care technologies (39). They can equally be used in the evaluation of public health interventions.

Sensitivity Analysis
Sensitivity analysis will be undertaken to test whether plausible changes in the values of the main variables affect the results of the analysis e.g. the age of the care-giver – there may be differences between spouse care-givers and those adult offspring care-givers who are in employment, for example.

Secondary Cost-Utility Analysis
We will conduct a cost utility analysis using EQ-5D to calculate QALYs (1) for carers and (2) for carers and people with dementia, on an experimental additive basis, where EQ-5D may have to be completed by proxy for people with dementia (45, 46). The addition of EQ-5D to the interview schedules for both care-givers and people with dementia allows us to undertake a secondary, more methodologically experimental, analysis which could measure and potentially combine the health utility gains to both people with dementia and their carers. This is in accord with the recommendation from the National Institute for Health and Clinical Excellence (NICE) that utility measures be included in trials of new drugs and interventions to facilitate cost per QALY calculation and that analysts consider the health effects of an intervention regardless of by whom they are accrued "For the reference case, the perspective on outcomes should be all direct health effects whether for patients or, where relevant, other individuals (principally carers)." (48, p.22). The potential impact on cost per QALY ratios in future, if health utility gains of carers were to be added to those of people with conditions such as dementia, has been recently highlighted (46). Given the findings of the trial platform, we consider that the costs of reminiscence therapy are not likely to be substantial, and the

effects may well be modest, which could result in a cost per QALY ratio with a large standard error. This, taken together with our concerns about the use of a generic measure of quality of life with people with dementia, leads us to propose the utility analysis as secondary to the analysis of cost effectiveness.,

Cost consequence Analysis

Table 1: Costs and consequences

**Costs**	**Consequences**
Costs of reminiscence therapy programmes	General and dementia-specific health-related quality of life of participants
Costs of primary and secondary sector health service utilisation	Use of dementia medication
Reminiscence based maintenance	Quality of life of carers
	Carer stress
	Independent living in the community
	Residential care

The cost-consequence analysis is a variant of cost-effectiveness analysis in which the components of incremental costs and consequences (health outcomes) of alternative programmes are listed without aggregation. This will be used for a comparison of secondary outcome measures of participants in the intervention and control arms of the study at baseline, 3 months, and 10 months. The inclusion of a cost –consequence analysis in addition to the standard cost effectiveness and cost utility analysis will set out clearly in a transparent manner the range of costs and consequences resulting from reminiscence therapy. This will provide the range of evidence required by commissioners and policy makers responsible for funding and coordinating services.

Research Governance

The trial is sponsored by the University of Wales Bangor.

A Trial Steering Committee will be established with an independent chair and at least three other independent members, recruited from the UKCRC Dementias & Neurodegenerative Research Network (DeNDRoN) and the corresponding network in Wales, NEURODEM Cymru. By analogy with two trials currently funded by the NHS HTA Programme – COGNATE and FolATED – we shall create the Data Monitoring & Ethics Committee (DMEC) as a sub-committee of the TSC, so as to enhance continuity and make efficient use of expert scientific resources. The TSC will include user/carer representatives from the NEURODEM Cymru panel. The first TSC/DMEC meeting will be held in January 2008, followed by meetings in December 2008 and December 2009.

4. Project timetable and milestones

December 1st 2007	Project commences:<br>Trial Manager & Coordinator in post<br>Research Officers recruited.<br>MREC approval and initial R&D approvals obtained<br>Facilitator training begins.
February 1st 2008	LREC and R&D approvals in place
February / March 2008	Baseline assessments for first wave
April 2008	First wave of treatment groups<br>Recruitment = 192
July 2008	Post-treatment assessments for first wave
July / August 2008	Baseline assessments for second wave
September 2008	Second wave of treatment groups<br>Recruitment = 384
December 2008	Post-treatment assessments for second wave
January / February 2009	Baseline assessments for third wave
February 2009	10 month follow-up first wave
March 2009	Third wave of treatment groups<br>Recruitment = 576
June 2009	Post-treatment assessments for third wave
July 2009	10 month follow-up for second wave
January 2010	10 month follow up for third wave
March 31st 2010	Database closed
April /May 2010	Data analysis
November 30th 2010	Write-up of draft final report and draft paper complete

Expertise

Our team offers a multi-disciplinary approach, including expertise in clinical psychology, psychiatry, social work, mental health nursing, health economics and randomised trial methodology.

Bob Woods is a clinical psychologist, who has been developing and evaluating psychological approaches in dementia care, including reminiscence therapy, since 1977; he is amongst the pioneers of an evidence-based approach in this field, and is a co-author of three Cochrane systematic reviews. He led the trial platform from which this proposal has arisen, and will be responsible for the overall leadership and management of the project. He will manage the Trial Coordinator and the research staff at Bangor

Ian Russell is a public health researcher who specialises in designing and conducting pragmatic RCTs, and developing patient-assessed measures of health outcomes for RCTs. He has recently brought these perspectives back to Wales, notably as founding director of the North Wales Organisation for Randomised Trials in Health (N-WORTH – a trials unit recognised and funded by CRCC, specifically for HTA trials), and as Chair of the Methodological Network of CRCC. N-WORTH will support the proposed trial, both methodologically and technically. In particular N-WORTH will adapt its trial software and Standard Operating Procedures (SOPs) to the trial, and contribute to the technical training and supervision of all researchers. He will also oversee the statistical, design, randomisation and data management aspects of the project.

Martin Orrell is an old age psychiatrist, who in a joint paper with BW (40) set out a manifesto for developing a rigorous evidence-based approach to the evaluation of psychological approaches in dementia care, which has resulted in a number of Cochrane reviews and a recently published RCT of a cognitive stimulation approach in dementia (2), including a health economics evaluation (37). He will manage the researchers based in London, covering a population base in Essex, through the North East London Mental Health Trust, and South London, where the Memorial Hospital, Woolwich will be a second centre, having participated n the trial platform and previous RYCT projects.

Errollyn Bruce is a key member of the Bradford Dementia Group, who has been involved in a number of innovative dementia care projects, including the development and descriptive evaluation of RYCT (12), working closely with Age Exchange. She will manage the researchers based in Bradford, and will also lead on the treatment adherence aspects of the trial.

Rhiannon Tudor Edwards is the Founding Director of the UWB Centre for Economics & Policy in Health, the largest group of health economists in Wales. She specialises in the economic evaluation of public health and complex interventions (43). She will manage and work with the dedicated trial health economics research officer, analyse results and write the health economics article describing trial findings.

John Keady has been at the forefront of developments in nursing research in dementia care, and has contributed greatly to the understanding of the perspectives of both people with dementia and their family care-givers, and has been instrumental in the development of relationship-centred care. In his new post at the University of Manchester, he is linked closely with clinical services in Bolton, Salford and Trafford Mental Health NHS Teaching Trust, and will be able to guide the implementation of the project in these large centres of population. He will manage the researchers based in Manchester.

Esme Moniz-Cook is a clinical psychologist who has been a pioneer of psychosocial interventions, in a variety of settings including primary care and care homes. She brings access to the Yorkshire and Humberside area through her position in the Humber Mental Health Teaching NHS Trust. She will manage the researchers based in Hull and the East Riding of Yorkshire.

Pam Schweitzer OBE is a key collaborator with and consultant to the project. She has been for many years Director of Age Exchange, a reminiscence-based charity, which has developed great expertise in reminiscence work with people with dementia and initiated the Remembering Yesterday Caring Today project, which led to the joint reminiscence groups being evaluated here. She has published extensively on this topic (41), and been a key-note speaker at many national and international conferences. She established the European Reminiscence Network, and since her retirement from Age Exchange, works in developing the field further through this network. She will oversee the training of facilitators and contribute to the quality assurance of the treatment groups.

Service users

Service users have already been involved in discussions of this proposal. Following the completion of the joint reminiscence groups and the reminiscence alone groups in Bangor as part of the MRC trial platform, the participants (people with dementia and care-givers) met with the PI (Bob Woods) and their recommendations for future work were sought. They were generally very positive about the groups, and were keen to know the results. From their perspective the benefits were very clear, and they were keen for the NHS locally to fund similar projects. They recommended that meetings should continue monthly after the 12 weekly sessions, to maintain the momentum. They appreciated and enjoyed being able to re-watch, on video, clips from the sessions. In addition, the user-carer research steering group at the Centre for Mental Health & Ageing, Humber Mental Health Teaching NHS Trust have perused the proposal and expressed their support for it.

We would intend to involve service users in the course of the project through NEURODEM Cymru (the Wales Dementias and Neurodegenerative Diseases Research Network). This would involve the appointment of several service users from the NEURODEM panel to monitor the project and advise the project team. This has been most useful in relation to other recent projects e.g. with the Alzheimer’s Society providing monitors for a trial of cognitive rehabilitation at University of Wales Bangor.

8. References

1. Brodaty H, Green A, Koschera A. Meta-analysis of psychosocial interventions for caregivers of people with dementia. Journal of American Geriatrics Society 2003;51:657-664.
2. Spector A, Thorgrimsen L, Woods B, Royan L, Davies S, Butterworth M, et al. Efficacy of an evidence-based cognitive stimulation therapy programme for people with dementia: randomised controlled trial. British Journal of Psychiatry 2003;183:248-254.
3. McShane R, Keene J, Gedling K, Fairburn C, Jacoby R, Hope T. Do neuroleptic drugs hasten cognitive decline in dementia? Prospective study with necropsy follow-up. British Medical Journal 1997;314:266-270.
4. Livingston G, Johnston K, Katona C, Paton J, Lyketsos CG. Systematic review of psychological approaches to the management of neuropsychiatric symptoms of dementia. American Journal of Psychiatry 2005;162(11):1996-2021.
5. Woods RT. Non-pharmacological techniques. In: Qizilbash N, editor. Evidence-based dementia practice. Oxford: Blackwell; 2002. p. 428-446.
6. Clare L, Moniz-Cook E, Orrell M, Spector A, Woods B. Cognitive rehabilitation and cognitive training for early-stage Alzheimer's disease and vascular dementia. In: The Cochrane Library. Chichester: Wiley; 2004.
7. Spector A, Orrell M, Davies S, Woods B. Reality orientation for dementia: a review of the evidence of effectiveness (Cochrane Review). In: The Cochrane Library. Oxford: Update Software; 1998.
8. DSDC-Wales. Services for people with dementia in Wales. Report No. 1: Residential and nursing home care in Wales. Bangor: Dementia Services Development Centre Wales; 2002.
9. Gibson F. The past in the present: using reminiscence in health and social care. Baltimore: Health Professions Press; 2004.
10. Woods RT, McKiernan F. Evaluating the impact of reminiscence on older people with dementia. In: Haight BK, Webster J, editors. The art and science of reminiscing: theory, research, methods and applications. Washington DC: Taylor & Francis; 1995. p. 233 - 242.
11. Woods B, Spector A, Jones C, Orrell M, Davies S. Reminiscence therapy for people with dementia (review). In: The Cochrane Database of Systematic Reviews. Chichester: Wiley; 2005.
12. Bruce E, Gibson F. Remembering Yesterday, Caring Today: Evaluators' Report. London: Age Exchange; 1998.
13. Thorgrimsen L, Schweitzer P, Orrell M. Evaluating reminiscence for people with dementia: a pilot study. The Arts in Psychotherapy 2002;29:93-97.
14. Tuokko H, MacCourt P, Heath Y. Home alone with dementia. Aging & Mental Health 1999;3:21-27.
15. Homer C. Using the Zelen design in randomized controlled trials: debates and controversies. Journal of Advanced Nursing 2002;38:200-207.
16. Folstein MF, Folstein SE, McHugh PR. 'Mini Mental State': a practical method for grading the cognitive state of patients for the clinician. Journal of Psychiatric Research 1975;12:189 - 198.
17. Logsdon RG, Gibbons LE, McCurry SM, Teri L. Assessing quality of life in older adults with cognitive impairment. Psychosomatic Medicine 2002;64:510-519.
18. Agar S, Moniz-Cook E, Orbell S, Elston C, Wang M. Measuring the outcome of psychosocial intervention for family caregivers of dementia sufferers: a factor analytic study. Aging & Mental Health 1997;1(2):166 - 175.
19. Goldberg DP, Hillier VF. A scaled version of the General Health Questionnaire. Psychological Medicine 1979;9:139-145.
20. Yesavage JA, Brink TL, Rose TL. Development and validation of a geriatric depression scale: a preliminary report. Journal of Psychiatric Research 1983;17:37 - 49.
21. Spruytte N, Van-Audenhove C, Lammertyn F, Storms G. The quality of the caregiving relationship in informal care for older adults with dementia and chronic psychiatric patients. Psychology & Psychotherapy: Theory, Research & Practice 2002;75:295-311.
22. Gallagher-Thompson D, Dal-Canto PG, Darnley S, Basilio LA, Whelan L, Jacob T. A feasibility study of videotaping to assess the relationship between distress in Alzheimer's disease caregivers and their interaction style. Aging & Mental Health 1997;1(4):346 - 355.
23. Wenger GC, Woods B, Keady J, Scott A, Devakumar M. Dementia Action Research & Education: older people with dementia and their carers: professional intervention in primary care: Final Report to Department of Health. Bangor: University of Wales Bangor; 2000.
24. American-Psychiatric-Association. Diagnostic and statistical manual of mental disorders (4th edition). Washington: American Psychiatric Association; 1994.
25. Hughes CP, Berg L, Danziger WL, Coben LA, Martin RL. A new clinical scale for the staging of dementia. British Journal of Psychiatry 1982;140:566-572.
26. Pattie AH, Gilleard CJ. Manual for the Clifton Assessment Procedures for the Elderly (CAPE). Sevenoaks: Hodder & Stoughton Educational; 1979.
27. Woods RT, Wills W, Higginson I, Hobbins J, Whitby M. Support in the community for people with dementia and their carers: a comparative outcome study of specialist mental health service interventions. International Journal of Geriatric Psychiatry 2003;18:298-307.
28. Thorgrimsen L, Selwood A, Spector A, Royan L, de-Madariaga-Lopez M, Woods RT, et al. Whose quality of life is it anyway? The validity and reliability of the Quality of Life - Alzheimer's Disease (QoL-AD) Scale. Alzheimer Disease and Associated Disorders 2003;17(4):201-208.

29. Hoe J, Hancock GA, Livingston G, Orrell M. Quality of life of people with dementia in residential care homes. British Journal of Psychiatry 2006;188:460-464.
30. Donaldson C, Tarrier N, Burns A. Determinants of carer stress in Alzheimer's disease. International Journal of Geriatric Psychiatry 1998;13(4):248 - 256.
31. Marriott A, Donaldson C, Tarrier N, Burns A. Effectiveness of cognitive-behavioural family intervention in reducing the burden of care in carers of patients with Alzheimer's disease. British Journal of Psychiatry 2000;176:557-562.
32. Kopelman MD, Wilson BA, Baddeley A. The Autobiographical Memory Interview. Bury St Edmunds: Thames Valley Test Company; 1990.
33. Alexopoulos GS, Abrams RC, Young RC, Shamoian CA. Cornell Scale for Depression in Dementia. Biological Psychiatry 1988;23:271-284.
34. Shankar KK, Walker M, Frost D, Orrell MW. The development of a valid and reliable scale for rating anxiety in dementia (RAID). Aging & Mental Health 1999;3(1):39-49.
35. Flint AJ, Rifat SL. Validation of the Hospital Anxiety and Depression Scale as a measure of severity of geriatric depression. International Journal of Geriatric Psychiatry 1996;11:991-994.
36. Beecham J, Knapp M. Costing psychiatric interventions. In: Thornicroft G, Brewin C, Wing J, editors. Measuring mental health needs. London: Gaskell; 1992. p. 163-183.
37. Knapp M, Thorgrimsen L, Patel A, Spector A, Hallam A, Woods B, et al. Cognitive stimulation therapy for people with dementia: cost-effectiveness analysis. British Journal of Psychiatry 2006;188:574-580.
38. Netten A, Curtis L. Unit costs of health and social care. Canterbury: Personal Social Services Research Unit, University of Kent; 2004.
39. Fenwick E, O'Brien BJ, Briggs A. Cost-effectiveness acceptability curves - facts fallacies and frequently asked questions. Health Economics 2004;13:405-415.
40. Orrell M, Woods RT. Tacrine and psychological therapies in dementia - no contest? International Journal of Geriatric Psychiatry 1996;11:189 - 192.
41. Schweitzer P, editor. Reminiscence in dementia care. London: Age Exchange; 1998.
42. NICE-SCIE. Dementia: supporting people with dementia and their carers in health and social care. Clinical Guideline 42. 2006.
43. Edwards RT, Ó Céilleachair AJ, Bywater T, Hughes D, & Hutchings J. A Parenting Programme for Children at risk of developing Conduct Disorder: A Cost-Effectiveness Analysis, BMJ 2007 in press.
44. Department of Health (2005). http://www.dh.gov.uk/PublicationsAndStatistics/Publications/PublicationsPolicyAndGuidance/PublicationsPolicyAndGuidanceArticle/fs/en?CONTENT_ID=4105545&chk=znAfqu
45. Bell CM, Araki, SS & Neumann PJ. The Association between care-giver burden and care-giver Health related Quality of Life, in Alzheimer Disease. Alzheimer Dis. Assoc. Disord. 2001; 15: 129 -136.
46. Dixon S, Walker M & Salek S. Incorporating carer effects into economic evaluation. Pharmaeconomics 2006: 24: 39 – 41.
47. Mistry H, Buxton M, Longworth L, Chatwin J and Peveler R on behalf of the AHEAD research team. Comparison of general practioner records and patient self-report questionnaires for the estimation of costs. Eur J Health Econom. 2005; 50: 261-266.
48. NICE. Guide to the Methods of Technology Appraisal. London: NICE; 2004.
49. http://www.euroqol.org/
50. Bucks RS, Ashworth DL, Wilcock GK, Siegfried K. Assessment of activities of daily living in dementia: development of the Bristol Activities of Daily Living Scale. Age & Ageing 1996; 25:113-120.

Appendices

1. Results from Trial Platform

2. Letter of support from Chair of Dementias Clinical Study Group, DeNDRoN UK

3. CONSORT Diagram for the proposed trial

4. MRC Trial Platform CONSORT Diagram

Appendix 1: Results from Trial Platform

	Baseline Reminiscence	Baseline Treatment as Usual	Post-treatment Reminiscence	Post-treatment Treatment as usual	Effect size
QoL-AD (patient-rated)	37.47 (5.46)	35.50 (5.33)	37.70 (5.22)	34.83 (5.84)	0.17
Relatives Stress Scale	22.56 (13.77)	20.50 (13.39)	21.49 (12.77)	24.33 (11.50)	0.36
GHQ - 28	19.97 (9.94)	21.82 (10.48)	20.19 (10.66)	27.64 (11.44)	0.56
GDS	2.95 (3.45)	3.09 (2.88)	3.08 (3.22)	5.09 (4.93)	0.56
AMI	69.01 (23.83)	72.86 (27.96)	67.58 (29.73)	58.14 (30.54)	0.54
QoL-AD (carer-rated)	30.82 (5.82)	30.35 (4.71)	30.99 (6.37)	27.60 (4.97)	0.50

Table 1: Comparison of mean scores (sd's) of participants attending reminiscence groups (N=38) with those receiving treatment as usual (N=12). Effect size = mean difference in change score / standard deviation of baseline sample.

	Baseline Reminiscence	Baseline Treatment as Usual	Follow-up Reminiscence	Follow-up Treatment as usual	Effect size
QoL-AD (patient-rated)	37.08 (5.38)	35.36 (5.57)	35.49 (4.99)	31.64 (11.79)	0.40
Relatives Stress Scale	20.11 (12.98)	20.50 (13.39)	22.78 (12.63)	27.33 (13.85)	0.31
GHQ - 28	18.97 (10.25)	22.00 (10.01)	21.14 (11.55)	30.33 (13.24)	0.62
GDS	2.46 (2.98)	3.09 (2.88)	3.41 (2.85)	5.64 (4.70)	0.48
AMI	70.01 (23.30)	72.86 (27.96)	58.94 (28.96)	58.59 (35.18)	0.13
QoL-AD (carer-rated)	30.96 (5.56)	29.59 (5.13)	30.11 (6.50)	26.82 (5.65)	0.33

Table 2: Comparison of mean scores (sd's) of participants attending reminiscence groups (N=35) with those receiving treatment as usual (N=10). Effect size = mean difference in change score / standard deviation of baseline sample.

Appendix **2: Letter of support from Chair of Dementias Clinical Study Group, DeNDRoN UK**

Telephone: 0161-291-5887
Fax: 0161 291 5882
Email: alistair.burns@manchester.ac.uk

DeNDRoN
North West
Dementias & Neurodegenerative Diseases Research Network

AB/SW
23 January 2007

Professor Robert Woods
Professor of Clinical Psychology of Older People
DSDC, Wales
Ardudwy
University of Wales Bangor
Holyhead Road
Bangor LL57 2PX

Dear Bob

Re: HTA trial - Reminiscence therapy in dementia

Thank you for contacting me about the above project.

With regard to adoption of the project by DeNDRoN, the Network cannot fully commit to supporting studies at this early stage and prior to review by an adoption panel. However, the study would be adopted by DeNDRoN if it met the following criteria:

Funding by the study has been awarded in national open competition
The study is designed for patient benefit
There are no significant issues with the population studied
There are no methodologicial issues that may impact upon recruitment
The study has gained appropriate Ethics Committee approval

With regard to your specific comment about working with Clinical Studies Group (CSG), we are, as you know, going to look in detail at specific projects. As chair of the group, I can assure you that we would ensure that members of the CSG worked closely with you on the project.

Please let me know if you need any further information.

With best wishes

Yours sincerely

Alistair Burns
Professor of Old Age Psychiatry
Associate Director, DeNDRoN
Chair, Clinical Studies Group in Dementia

Appendix 3: CONSORT diagram

Flow diagram for proposed trial

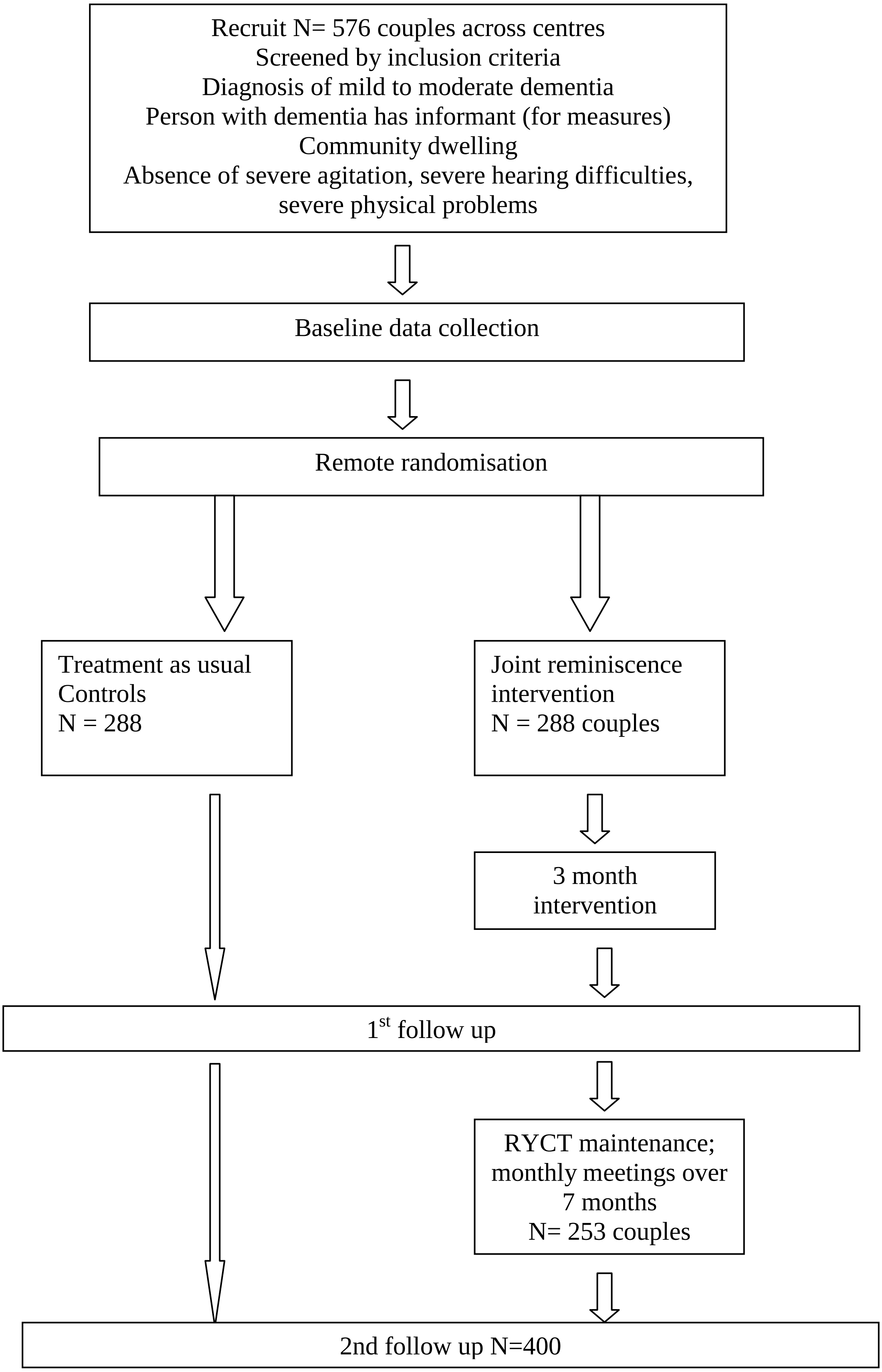

Appendix 4

MRC Trial Platform CONSORT diagram

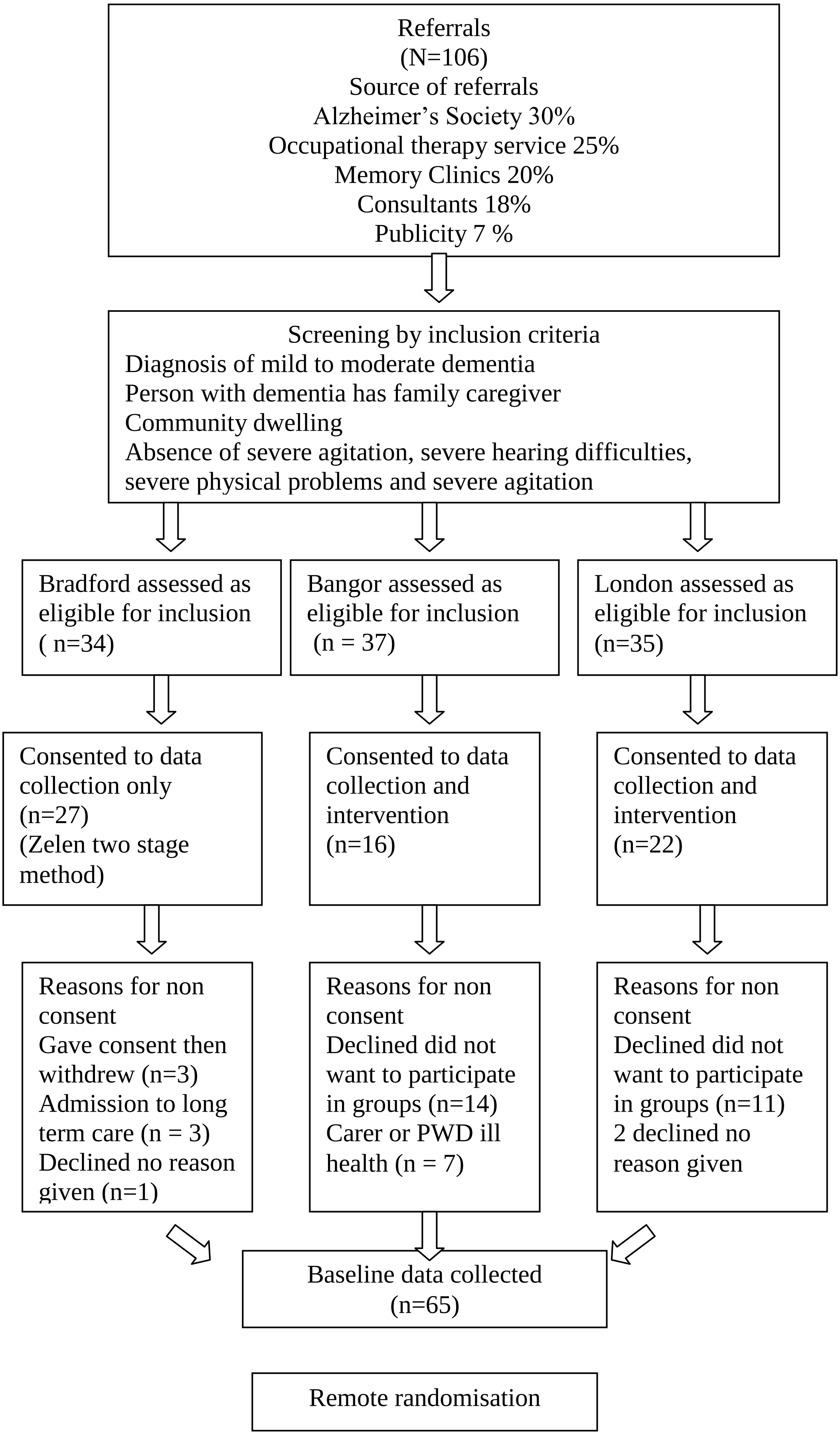

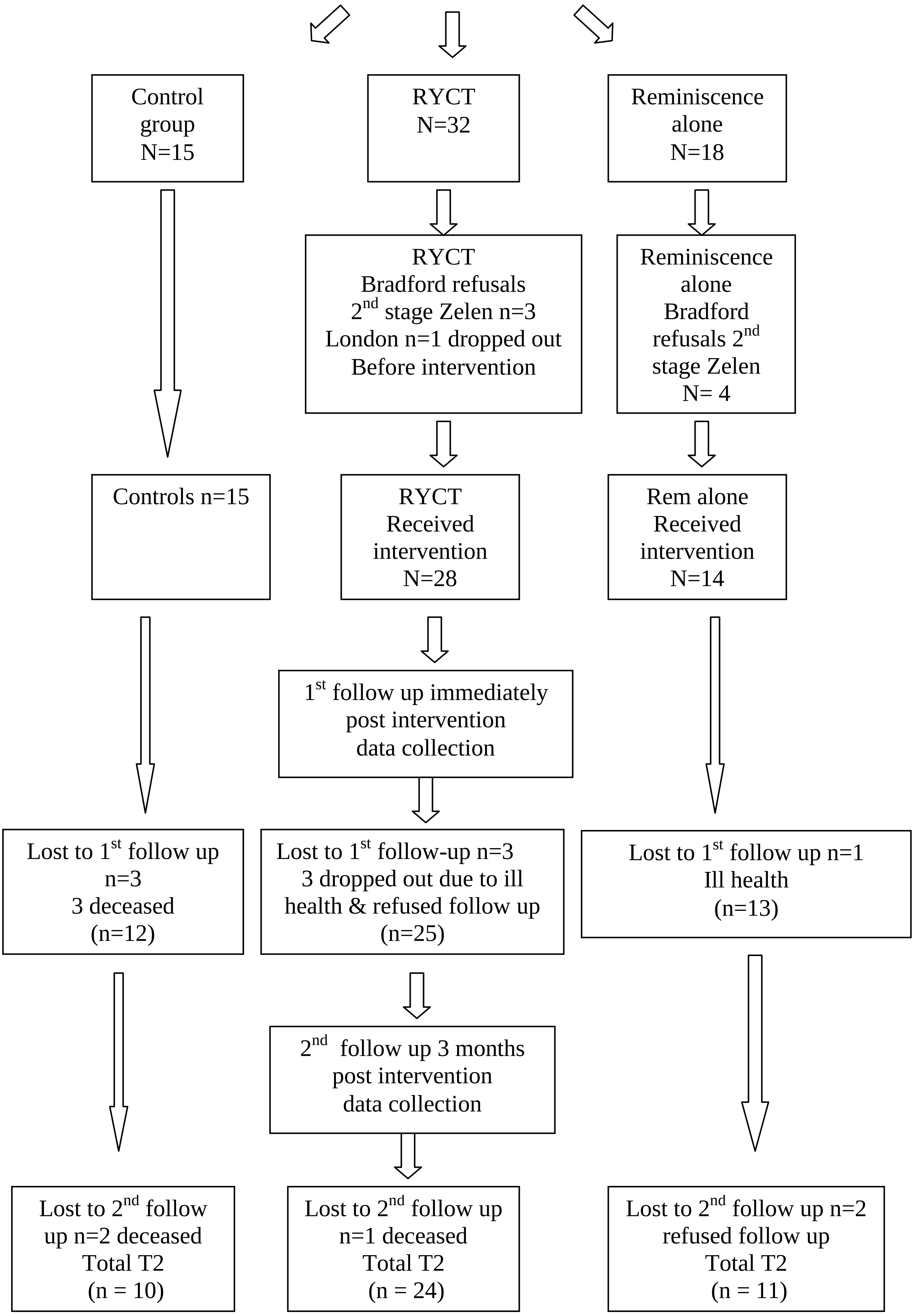
Control group N=15
RYCT N=32
Reminiscence alone N=18
RYCT Bradford refusals 2nd stage Zelen n=3 London n=1 dropped out Before intervention
Reminiscence alone Bradford refusals 2nd stage Zelen N= 4
Controls n=15
RYCT Received intervention N=28
Rem alone Received intervention N=14
1st follow up immediately post intervention data collection
Lost to 1st follow up n=3 3 deceased (n=12)
Lost to 1st follow-up n=3 3 dropped out due to ill health & refused follow up (n=25)
Lost to 1st follow up n=1 Ill health (n=13)
2nd follow up 3 months post intervention data collection
Lost to 2nd follow up n=2 deceased Total T2 (n = 10)
Lost to 2nd follow up n=1 deceased Total T2 (n = 24)
Lost to 2nd follow up n=2 refused follow up Total T2 (n = 11)
